# Regioselective Dimerization of Methylcyclopentadiene inside Cucurbit[7]uril

**DOI:** 10.1002/chem.202403964

**Published:** 2025-02-05

**Authors:** Khaleel I. Assaf, Foad N. Tehrani, Guillermo E. Quintero, Robert Hein, Margarita E. Aliaga, Werner M. Nau

**Affiliations:** ^1^ Department of Chemistry Faculty of Science Al-Balqa Applied University 19117 Al-Salt Jordan; ^2^ School of Science Constructor University Campus Ring 1 28759 Bremen Germany; ^3^ Facultad de Química y de Farmacia Escuela de Química Pontificia Universidad Católica de Chile Casilla 306 Santiago 6094411 Chile

**Keywords:** Host-Guest Complexes, Macrocycles, Regioisomers, Rate Acceleration, Supramolecular Chemistry

## Abstract

The molecular confinement within rigid macrocyclic receptors can trigger catalytic activity and steer the selectivity of organic reactions. In this work, the dimerization of methylcyclopentadiene (MCPD) isomers in the presence of cucurbit[7]uril (CB7) was found to display, besides a large rate acceleration, a striking regioselectivity in aqueous solution at pH 3, different from the products predominating in the absence of the supramolecular catalyst. Among the different possible regioisomers and diastereomers, the *endo*‐3,7‐dimethyl‐3a,4,7,7a‐tetrahydro‐1H‐4,7‐methanoindene adduct was selectively formed, which is otherwise found only as a minor product in the dimerization of neat MCPD or in commercial dimeric mixtures. This product originates from the reaction of the heteroternary complex of 1‐MCPD and 2‐MCPD within CB7, in which the methyl groups are positioned in an “anti‐diaxial” arrangement and point towards the open portals of the macrocycle, resulting in a preferred packing of the reacting cyclopentadiene rings. The selectivity of the dimerization of MCPD in the absence and presence of CB7 is supported by quantum‐chemical calculations.

## Graphical abstract

Cucurbit[7]uril serves as a supramolecular catalyst for the cycloaddition reaction of methylcyclopentadiene, affording a 10^4^‐fold rate enhancement and a significant change in the regioselectivity. This reaction predominantly yields a single product that is both kinetically and thermodynamically disfavored and typically observed only as a minor component in the dimerization of neat methylcyclopentadiene or in commercial dimeric mixtures.

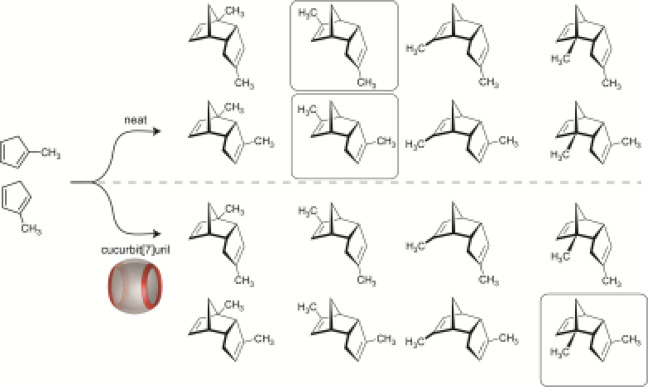

## Introduction

Enzymes are natural catalysts that promote biochemical reactions by utilizing their specific active binding sites. By analogy, macrocycles have emerged as functional supramolecular catalysts that mimic the natural ones in regard to their concave binding pockets.[^1^, ^2^, ^3^, ^4^, ^5^, ^6^, ^7^, ^8^] The macrocyclic microenvironment can enhance reaction rates and lead to new selectivity patterns that cannot be obtained in bulk solution.[^9^, ^10^, ^11^, ^12^, ^13^, ^14^, ^15^] In aqueous solution, cyclodextrins,[^16^, ^17^] cucurbiturils,[^18^, ^19^, ^20^, ^21^, ^22^] and calixarenes[^23^, ^24^] have been successfully implemented as macrocycles in supramolecular catalysis, even if product inhibition has frequently limited catalytic turnover.[^1^, ^12^, ^25^, ^26^, ^27^, ^28^, ^29^, ^30^, ^31^, ^32^, ^33^, ^34^, ^35^]

Cucurbit[*n*]urils (CB*n*) have been widely employed as supramolecular catalysts for unimolecular and bimolecular reactions in aqueous solution.[^3^, ^19^, ^36^, ^37^] The first example dates back to 1983, in which Mock and coworkers reported that CB6 catalyzed the [3+2] cycloaddition between two guests with azide and acetylene groups.^[12]^ Subsequently, several cycloaddition and photodimerization reactions have been explored.[^21^, ^35^, ^38^, ^39^, ^40^, ^41^] Among the CB*n* homologues, CB8 is well‐known to mediate bimolecular reactions (mostly photodimerization reactions),[^19^, ^36^, ^37^, ^39^, ^40^, ^42^] which is attributed to its large cavity size (367 Å^3^) that can accommodate two guest molecules and form ternary complexes. In contrast, the smaller CB*n* homologues (CB6 and CB7) have been explored to a lesser extent, and not for pure hydrocarbon substrates.[^12^, ^34^, ^40^, ^41^, ^43^, ^44^, ^45^]

**Figure 1:**
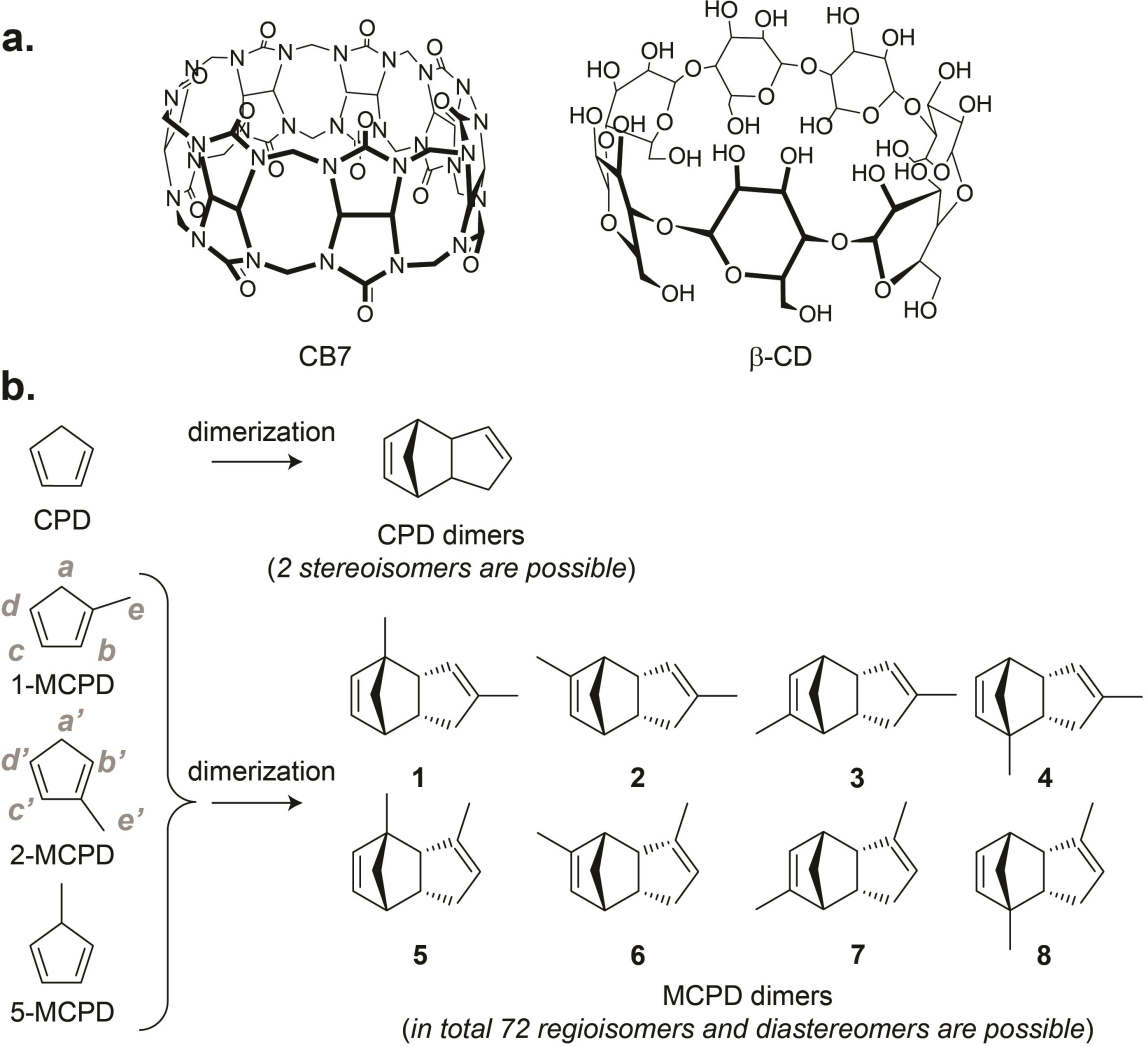
Chemical structures of **a**. cucurbit[7]uril (CB7) and β‐cyclodextrin (β‐CD), **b**. cyclopentadiene (CPD), the methylcyclopentadiene isomers (MCPD), and their corresponding dimerization product(s); major products for MCPD (**1**‐**8**) are shown.

Recently, we observed a large rate acceleration (*k*_cat_/*k*_uncat_ ca. 4×10^5^ M) for the cyclopentadiene (CPD) dimerization inside CB7 (Figure 1a), approaching the theoretically predicted maximum for exactly this reaction.^[34]^ However, the *endo* selectivity of the Diels‐Alder reaction remained unaffected inside CB7. To investigate the effect of this highly efficient supramolecular catalyst on the regioselectivity of this cycloaddition, we have now studied the dimerization of methylcyclopentadiene (MCPD, Figure 1b) inside CB7, which exists as a mixture of three positional isomers;[^46^, ^47^, ^48^] in principle, MCPD dimerization can give rise to 72 regioisomers and diastereomers, among which at least 8 (**1**‐**8**) are observed in the dimerization of the neat diene (Figure 1).[^46^, ^47^, ^48^, ^49^, ^50^, ^51^, ^52^] Indeed, we observed a pronounced change in the regioselectivity of this cycloaddition in the presence of CB7, which now affords predominantly one product, *endo*‐3,7‐dimethyl‐3a,4,7,7a‐tetrahydro‐1H‐4,7‐methanoindene (**8**), the one which is not predominant when the reaction is allowed to proceed in neat solution and the one which is not predominant in commercial MCPD.

## Results and Discussion

**Figure 2:**
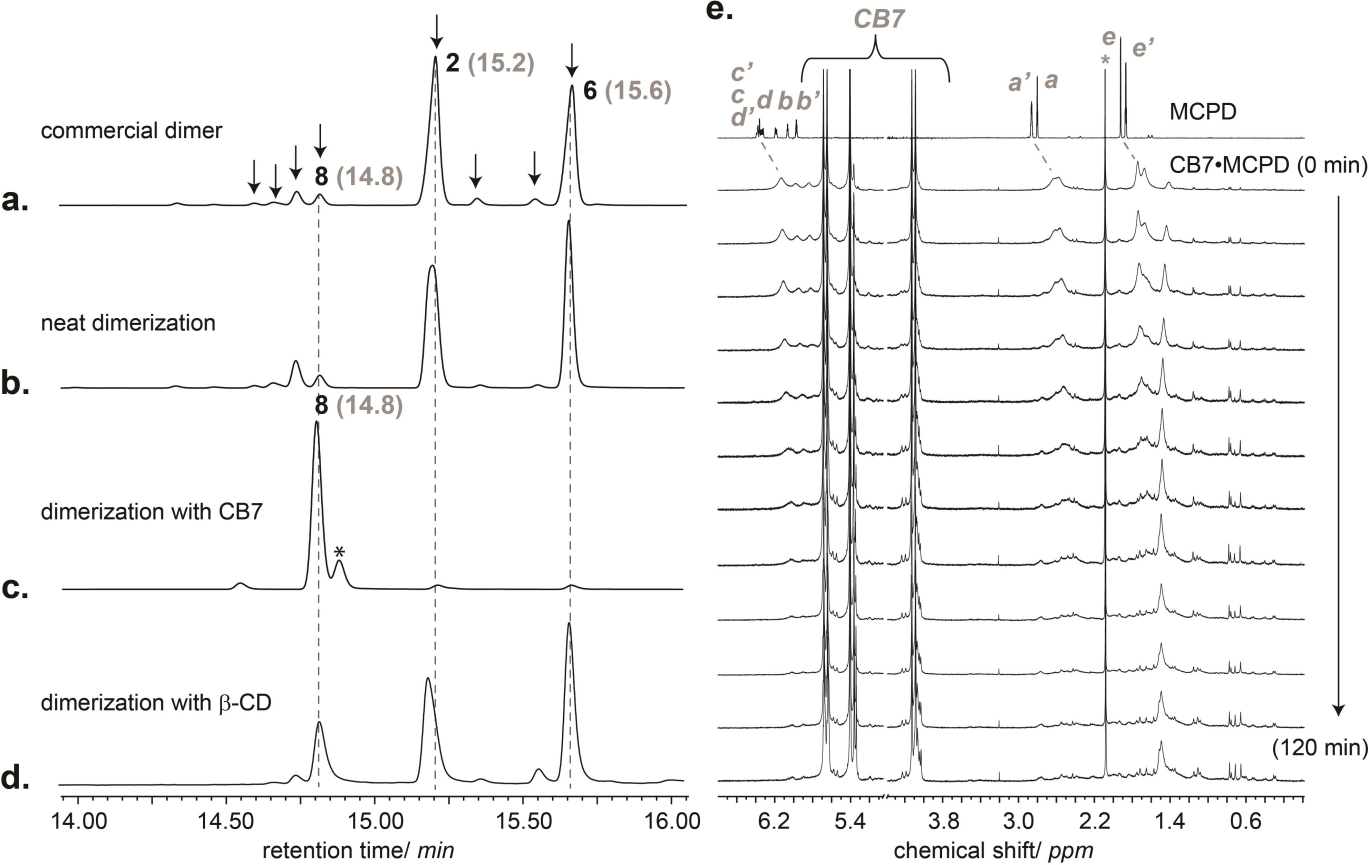
**a.‐d**. GC‐FID chromatograms for **a**. commercial MCPD dimer and the dimerization products (the chemical structures of the 8 main dimers are given in Figure 1b) of **b**. in the neat liquid and inside the macrocyclic cavity of **c**. CB7 and **d**. β‐CD (3 mM of host and 6 mM MCPD, pH 2.8; products were extracted with organic solvent). Note the entirely new product peak of unidentified origin in the presence of CB7 (marked with *). **e**. Dimerization of MCPD (40 mM) in the presence of CB7 (3 mM), monitored by ^1^H NMR in D_2_O, pD ~3; see Figure 1b for peak assignment.

As is the case for CPD, MCPD undergoes thermal Diels‐Alder dimerization and is accordingly commercially available in dimeric form. It needs to be thermally “cracked” to its monomeric isomers (1‐, 2‐, and 5‐MCPD, Figure 1b),^[51]^ which can be stored for a few days when kept refrigerated. The first two monomers are predominant (>99 %), and can undergo isomerization via sigmatropic [1,5]‐hydrogen shift at the pyrolysis temperature of the MCPD dimers,[^51^, ^53^] which makes it difficult to isolate and study the reactivity of each individual isomer separately. Commercial MCPD shows 8 distinct peaks when analyzed by gas chromatography (GC).^[54]^ They have been previously assigned as the regioisomeric products **1**–**8** (Figure 2a) by GC, IR, and NMR spectroscopy,[^46^, ^47^, ^48^, ^49^, ^50^, ^51^, ^52^] among which dimers **2** and **6** are by far dominant (ca. 90 %). When MCPD monomers were allowed to dimerize as neat liquid at ambient temperature, we obtained the same 8 products (Figure 2b), with only minor variations in relative percentages, likely due to the different dimerization conditions of the commercial sample. When the reaction of MCPD (6 mM) was carried out in aqueous solution in the presence of CB7 (3 mM, half an equivalent, as the reactant is expected to form and react as a 1 : 2 host‐guest complex, see isothermal titration calorimetry results below), at pH 2.8‐3 (due to acid traces in the CB7 sample from synthesis),^[55]^ product **8** was found to be the main product (Figure 2c), while the others, in particular the two dimers dominant in the neat reaction, became minor products. In comparison, when the reaction was performed in the presence of β‐cyclodextrin (β‐CD), an alternative macrocycle with similar inner cavity size as CB7, much smaller variations in product composition were observed, in the content order **2**,**6** ≫ **8** ≫ others (Figure 2d). It should be noted that the reaction of MCPD in the presence of CB7 showed also a reaction rate acceleration, albeit less pronounced than for the dimerization of CPD inside CB7.^[34]^ In the presence of CB7, almost full conversion of MCPD was observed after 17 h, while dimerization of MCPD as neat liquid or in water (pH 3) as solvent showed only 54 % and 17 % conversion, respectively. Accordingly, the reaction of MCPD showed a pronounced rate acceleration for dimerization with a high selectivity for the formation of product **8**. The estimated rate constant (*k*) for the second order reaction of MCPD dimerization, based on the GC‐conversion after 17 h, revealed that neat MCPD dimerizes slowly, with a rate of ca. 3×10^−7^ M^−1^ s^−1^, which is slightly slower than for the parent CPD in its neat form (9×10^−7^ M^−1^ s^−1^).^[34]^ In the presence of CB7 at pH 2.8, the (apparent) bimolecular reaction rate increased strongly, by more than a factor of 10^4^, when using the neat reactant as reference, as previously detailed for CPD.^[34]^ In addition, we have observed that the rate acceleration and change in regioselectivity for MCPD dimerization are diminished when the cavity of CB7 is blocked by a high‐affinity binder (e.g., adamantylamine), in accordance with the results of CPD.^[34]^

To analytically validate the identity of the main dimer of the CB7‐mediated cycloaddition and to compensate for drifts in the retention times across the chromatographic runs, we further quantified the linear retention indices of the products by using *n*‐alkanes as internal standard (Figure S2 in SI). Programmed‐temperature retention index calculations were applied according to van den Dool and Kratz.^[56]^ The main MCPD dimers eluted between the C_11_ and C_12_ *n*‐alkanes, with retention indices spanning between 1113 and 1181. These values are the same, within systematic drifts related to the choice of different GC columns, to previously reported retention indices (Table S1 in SI).[^49^, ^52^, ^57^] Further evidence was obtained by a GC co‐injection experiment, in which a sample of the CB7‐mediated reaction was combined with the commercial dimer sample as internal reference (Figure S2 in SI). The superimposed chromatograms indicated a significantly increased content of dimer **8**, supporting the retention index result and confirming the identity of the dimerization product inside CB7 as dimer **8**.

The formation of host‐guest inclusion complexes between CB7 and MCPD in aqueous solution could be conveniently established by ^1^H NMR spectroscopy, which also allowed for online monitoring of the reaction inside CB7 (Figure 2e). The MCPD ^1^H NMR signals^[58]^ experienced upfield shifts and significant broadening, indicative of host‐guest complex formation and the inclusion of MCPD inside the hydrophobic cavity of CB7. As the reaction progressed, the signals of MCPD disappeared and a new set of signals appeared in the aliphatic range of the NMR spectra (0.4–3.2 ppm), which correspond to the MCPD dimer (encapsulated inside CB7), indicating full conversion and revealing catalytic turnover. It should be noted that the reaction solution turned slightly turbid due to (partial) precipitation of the resulting dimer complex with CB7.^[59]^ This precipitation, and the complexity of the NMR spectra associated to the multiple CB7•product complexes, prevented a quantitative analysis of the complexation thermodynamics and product composition. The latter was more reliably performed by GC analysis after extraction of the dimer products with organic solvent (see above), while the former was investigated next by isothermal titration calorimetry (ITC).

**Figure 3:**
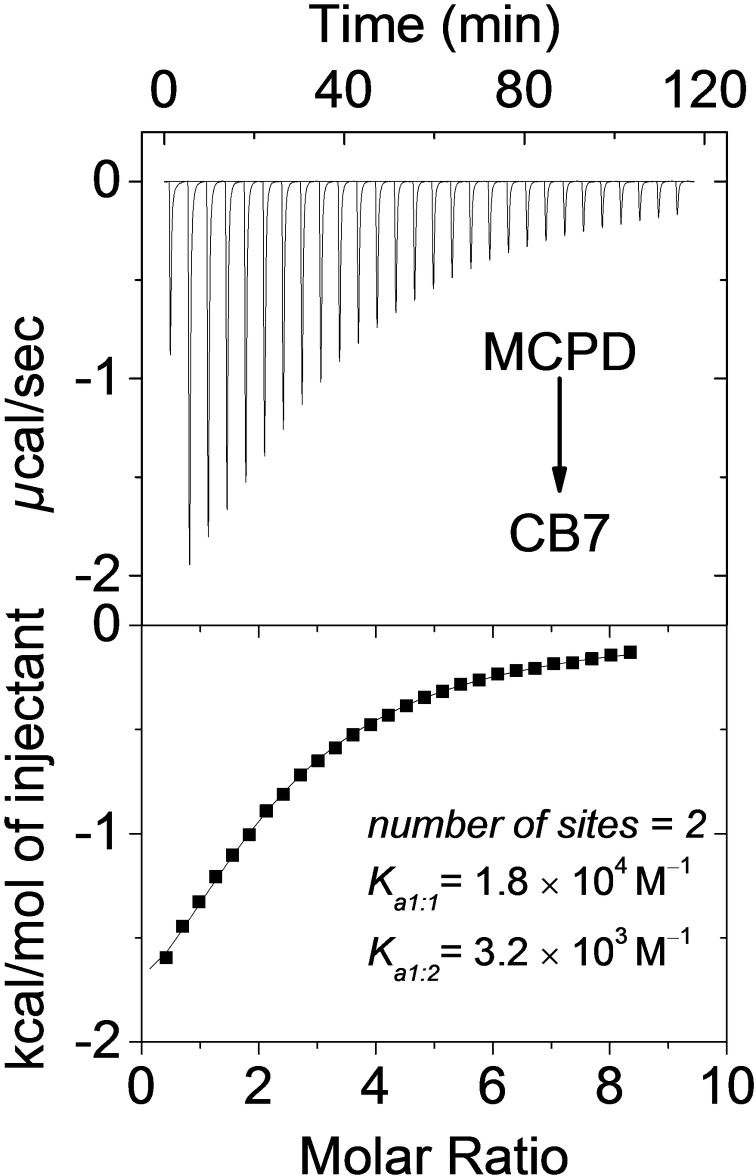
Raw ITC data for sequential twenty‐seven injections of a 3.8 mM MCPD solution injected into a CB7 solution (0.1 mM, top) and apparent reaction heats obtained from the integration of the calorimetric traces (bottom) at pH 7. Δ*H*_1:1_ and Δ*H*_1:2_ were −2.6±0.5 and −3.9±0.5 kcal/mol. The error in *K*_a_ values, extracted from a fitting according to a sequential binding site model, is 10 %.

ITC measurements (Figure 3) in water were possible, because the reaction of MCPD was pH‐dependent (see below) and sufficiently slow at pH 7 to reliably run the titrations. The thermograms confirmed the formation of 1 : 2 complexes and millimolar (and higher) binding constants according to a sequential binding model (*K*_a1:1_=1.8×10^4^ M^−1^, *K*_a1:2_=3.2×10^3^ M^−1^); these values should allow for a high population of the ternary complexes required for the macrocycle‐promoted cycloaddition at the selected millimolar concentrations (degree of complexation for 1 : 2 host‐guest complexation estimated as >75 %), as observed by ^1^H NMR and confirmed through the observed reactivity.

**Figure 4:**
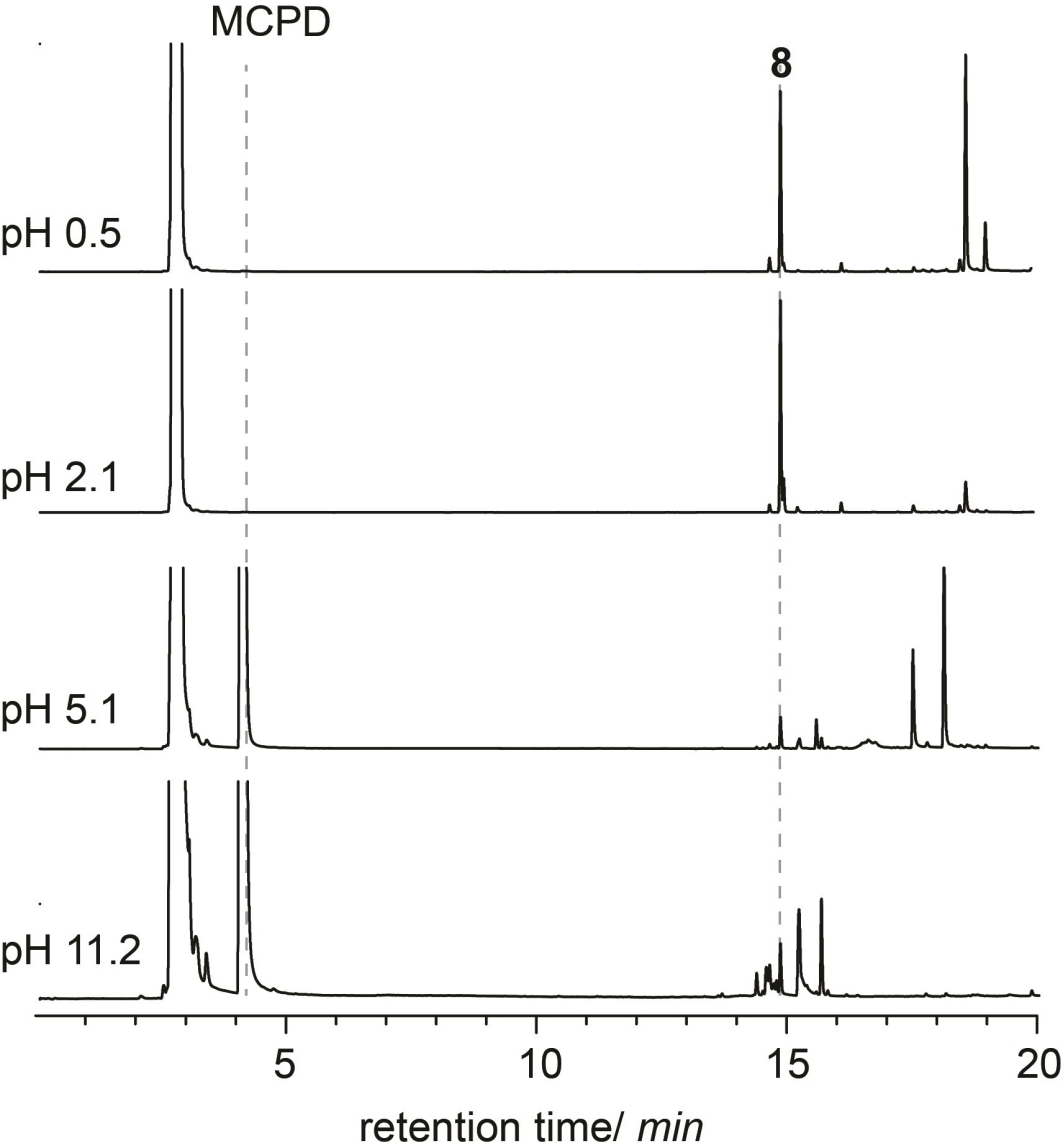
GC‐FID chromatograms for the dimerization of MCPD (6 mM) inside CB7 (3 mM) after 24 h at different pH values.

Interestingly, the dimerization reaction of MCPD inside CB7 was found to be pH‐dependent (Figure 4). This is in contrast to the parent CPD, for which the dimerization was found to be catalyzed by CB7 across a wide pH range.^[34]^ We observed a significant pH effect on the reaction rate and also on the selectivity. At strongly acidic pH values, below pH 1, compound **8** remained the major cycloaddition product, but we observed side reactions related to acid‐catalyzed reactions with water, leading to the formation of products with large retention times, presumably alcohols. Above pH 5, the reaction rate was greatly reduced, and the spectrum of cycloaddition products resembled more that observed in the absence of CB7. We could further exclude that the presence (and binding)^[60]^ of sodium cations was responsible for this change in selectivity in alkaline media (see Figure S3 in SI), suggesting that the catalysis by CB7 only took place in acidic solution. We speculate that the protonation of one carbonyl rim of CB7 (p*K*_a(CB7)_=2.2)[^60^, ^61^] introduces an asymmetry in the ternary complex, which has a favorable electronic effect on the cycloaddition reaction, while not principally modifying the reaction mechanism and retaining the product selectivity.

It is well known that cucurbituril macrocycles show preferential binding of certain constitutional isomers of hydrocarbons, commonly the more spherical or cavity space‐filling ones.[^62^, ^63^, ^64^, ^65^, ^66^, ^67^] For example, CB6 binds isobutane more strongly than *n*‐butane^[67]^ and CB7 binds *ortho*‐disubstituted benzenes, e. g., xylene, more tightly than its *para* and *meta* isomers.^[62]^ While the differential isomeric affinities can be employed for separation applications, the use in supramolecular catalysis of hydrocarbon substrates has not yet been reported.

**Figure 5:**
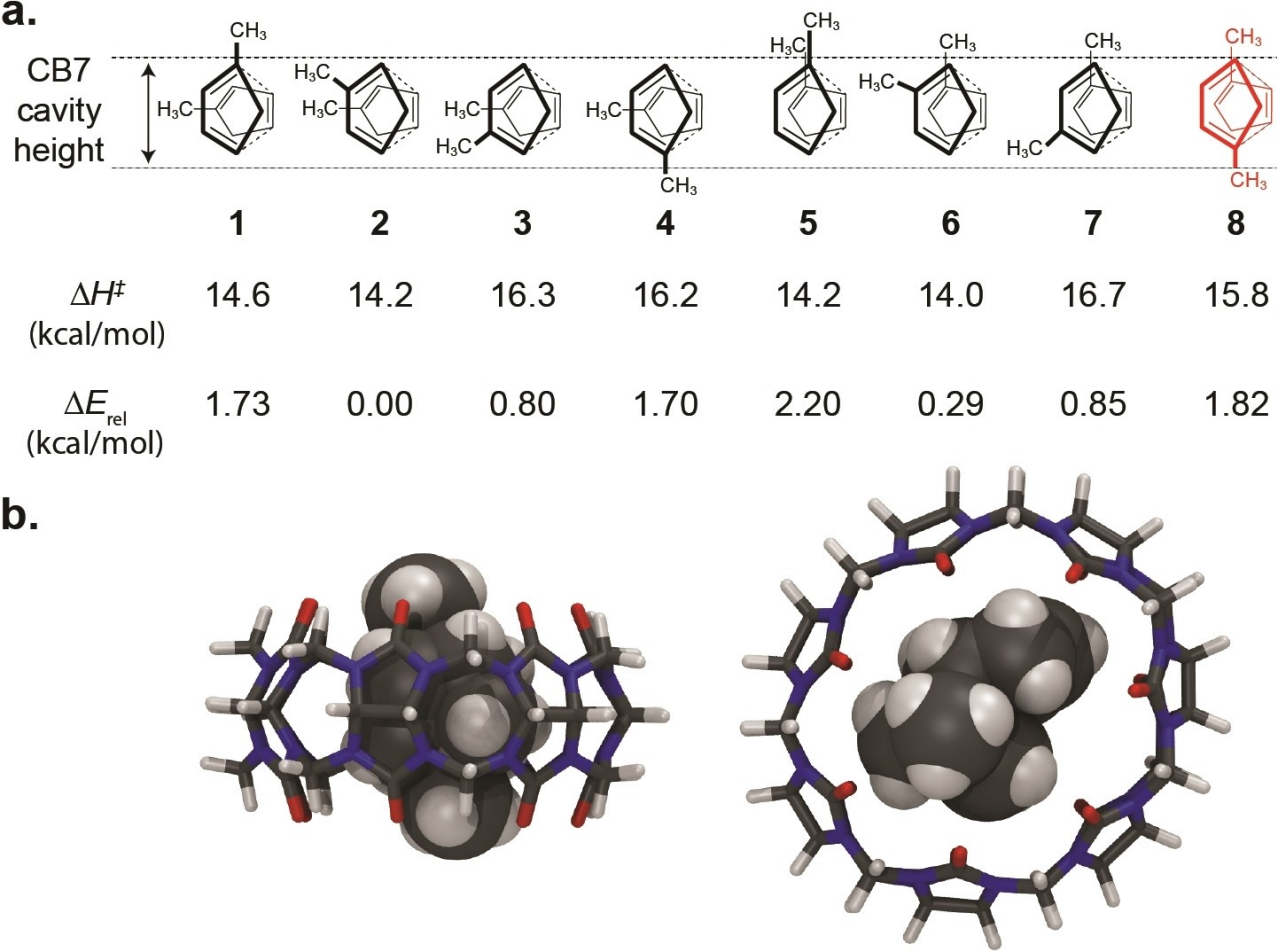
**a**. Schematic arrangement of the two MCPD reactants resembling the transition states for the dimerization leading to the 8 experimentally observed dimers **1**–**8**. The dashed lines indicate the upper and lower cavity boundaries of CB7, that is, all groups positioned in between the boundaries will increase the packing coefficient of the already tightly packed complexes. DFT‐calculated activation enthalpies of dimerization (Δ*H* ^≠^) and relative stabilities (Δ*E*_rel_) of the corresponding dimers are given below the structures (B3LYP−D3(BJ)/6‐31+G* level of theory). **b**. Space‐filling representations of the optimized structure (B3LYP−D3(BJ)/6‐31+G* level of theory) of the host‐guest inclusion complex of dimer **8** with CB7. Note the ideal packing of the diametrally opposed methyl groups protruding into the portal regions, which is reminiscent of the ultrahigh affinity complexes of disubstituted ferrocene^[69]^ and diamantane^[70]^ guests inside the same macrocycle.

In principle, 72 hydrocarbon products (regioisomers and diastereomers) can be formed by the cycloaddition of the three MCPD monomers. As is the case for the parent CPD, the *endo* rule is obeyed, such that only *endo* diastereomers (the kinetically favored products) are experimentally observed, eliminating 36 potential products. Moreover, due to reasons of alkene stability, the 1‐MCPD and 2‐MCPD positional isomers are the dominant monomeric constituents after cracking (>99 %),^[48]^ such that an additional 20 potential dimer structures (those involving 5‐MCPD) can be discarded. Lastly, among the remaining 16 possible products, 8 would involve reactions of an alkylated (electron‐rich) double bond in the dienophile, which is disfavored on electronic grounds. This leaves the experimentally observed dimers **1**–**8** as expected products. The relative distribution among these 8 products is less straightforward to predict with simple organic structure‐reactivity or product stability arguments, but density functional theoretical (DFT) calculations succeed in predicting that products **2** and **6** are indeed the kinetically and thermodynamically most stable ones, see activation enthalpies (Δ*H* ^≠^) and relative stabilities (Δ*E*_rel_) in Figure 5a; detailed thermodynamic data of the *endo* transition states are provided in the SI, Table S2. These are also the ones found to be most abundant in the commercial mixtures, the ones obtained by dimerization of neat MCPD, and the ones in the presence of β‐CD as supramolecular catalyst.

The regioselective formation of product **8** in the presence of CB7 in aqueous solution is interesting because it provides access to an otherwise practically elusive (only observed in trace amounts) and kinetically and thermodynamically less favored (according to DFT calculations, Figure 5a) product. This inversion in selectivity can only be explained through a pronounced effect of CB7 on the relative reaction rates for formation of products **2**, **6**, and **8**. Insight into the factors governing the inner‐phase dimerization can be obtained from the previously reported analysis of the Diels‐Alder reaction of CPD inside CB7, especially from the packing coefficient analysis. The molecular volume of CPD and MCPD is 76 and 93 Å^3^, respectively. Accordingly, the formation of a ternary complex composed of two CPD monomers encapsulated inside the CB7 cavity (inner volume: 242 Å^3^) gives rise to a tight packing coefficient of 63 %, significantly above the ideal packing coefficient of 55 %.^[34]^ The reduction of packing in the course of the Diels‐Alder reaction (from 63 % for the ternary (CB7•2CPD) monomer complex to 58 % for the CPD dimer complex) has been used to account for the large rate acceleration and catalytic effect of CB7 on this reaction.^[34]^ The preferred packing and expectedly strong binding of the dimer is also responsible for the observed product inhibition in the course of the reaction,^[34]^ which requires stoichiometric amounts (1 : 2 CB7:MCPD) to approach full conversion. It should be noted that the cavity size of smaller or larger CB*n* homologues, such as CB6 (142 Å^3^) and CB8 (367 ^3^), is either too small to accommodate two MCPD units or too large to induce a catalytic effect, respectively.^[34]^

If one inspects the relative orientations of the transition‐state structures leading to the different dimeric products **1**–**8**, almost all structures, except for structure **8** and **5**, require at least one methyl group to be positioned inside the CB7 cavity (located between the dashed lines in Figure 5a). This would result in a further increase in packing coefficient to 69 % (for **1**, **4**, **6**, and **7**) or even 77 % (for **2** and **3**), which would render the corresponding monomer geometries very difficult to achieve,^[19]^ that is, their formation rates would be effectively reduced. Only the transition states leading to products **8** and **5** would retain the same packing coefficient as for CPD, because the methyl groups could protrude outside the inner cavity into the empty portal regions of CB7. Among those two least tightly packed structures, the “*anti*‐diaxial” structure **8** will be obviously more favorable, because the two methyl groups would not cause steric clashes with each other and within the same portal region (Figure 5b). This was supported by DFT calculations (Figure S4), which indicated that dimer **8** forms the most stable inclusion complex with CB7. This *syn*/*anti* selectivity is reminiscent of the head‐to‐head *versus* head‐to‐tail dimer selectivity observed in photocycloadditions promoted mostly by the larger cucurbit[8]uril homologue, with the difference that the selectivity in these reaction is commonly steered through ion‐dipole interactions with attached ammonium or other cationic groups.[^39^, ^68^]

It is striking that the preferred cycloaddition product in the presence of CB7 can be reliably predicted alone from the preferred packing, even if the 8 tricyclic dimer structures show great shape resemblance and lack differences in functional groups as recognition features (Figure 1). Note further that product **8** originates from the reaction of the “heteroternary” complex, 1‐MCPD•2‐MCPD•CB7, not from a homoternary complex (such as dimer **6**). This may account for the fact that the reaction can swiftly proceed to almost complete conversion, because both monomers are being consumed simultaneously (their relative contribution in the monomer mixture is almost the same, 47 : 53).^[46]^ Interestingly, for β‐CD, a similar packing effect may be operational, although to a smaller degree, because the original products are less effectively suppressed (Figure 2d).

As a mechanistic complexity, it turned out that the catalytic effect of CB7, namely the reaction rate acceleration and the preferential formation of dimer **8**, was only observed in acidic solution, below pH 4. This suggested that the active species is not CB7 itself, but its protonated form, CB7•H^+^, which becomes populated in this pH range (p*K*_a(CB7)_=2.2).[^60^, ^61^] We presume that the presence of the proton at the portal of CB7 is also essential to promote the inner‐phase reaction of the heteroternary complex under formation of dimer **8**. Whether the action of the proton unfolds through a simple electrostatic effect or whether it involves a polar cycloaddition mechanism under formation of a tertiary allylic carbocation (which could be stabilized by CB7, as known for other carbocations),[^29^, ^71^, ^72^] is presently unknown and will require follow‐up studies. Note also that MCPD undergoes itself acid‐catalyzed (side) reactions with water at very low pH, which could also be traced back to the formation of such carbocation intermediates.

## Conclusions

The cavities of cucurbiturils offer a unique playground for supramolecular catalysis. While rate accelerations by these macrocycles have been intensively investigated,[^12^, ^13^, ^19^, ^34^, ^73^] comparably lesser attention has been paid to changes in the chemoselectivity of organic reactions induced by cucurbiturils.[^74^, ^75^] We have found that the cycloaddition of methylcyclopentadiene monomers, which generally produces 8 regioisomers in solution, with a preference for the formation of the two kinetically and thermodynamically most stable ones, yields a different major product in the presence of CB7. The dimer produced in the presence of CB7 is neither kinetically nor thermodynamically most favorable, but it is the one for which the transition state “packs best” inside the macrocyclic cavity, resulting in an impressive regioselectivity.

## Experimental Section

### Chemicals

Methylcyclopentadiene dimer (mixture of isomers, 93 %) was purchased from Sigma‐Aldrich. CB7 was synthesized according to ref. [55].

### Instrumentation

NMR spectra were recorded on a JEOL ECX 400 spectrometer. Chemical shifts (*δ*) are reported in parts per million (ppm) downfield from tetramethylsilane (TMS=0) or relative to CHCl_3_ (7.26 ppm) or HOD (4.79 ppm). Isothermal titration calorimetry (ITC) experiments were carried out on a VP‐ITC from Microcal, Inc., at 25 °C. GC‐FID (GC‐2010 Shimadzu) program: Vf‐1 ms column (30 m×0.25 mm); *T*_inj_=100 °C and *T*_det_=300 °C were always constant; oven temperature 30 °C (hold 5 min), subsequently ramp (10 °C/min) to 230 °C (hold at 230 °C for 10 min), MCPD (mixture of 1‐ and 2‐MCPD) retention time=4.10 min, MDCP dimer (major product with CB7, **8**) retention time=14.82 min.

### Thermal Cracking of MCPD Dimers

Using a distillation apparatus, 50 mL of commercial MCPD dimer in a 100 mL round‐bottomed flask were heated to 240–250 °C by using a sand‐bath under atmospheric pressure. The monomer was collected in a receiver flask placed in an ice bath. Subsequently, the MCPD monomer mixture (mainly 1‐ and 2‐MCPD) was further distilled at 140 °C by using an oil‐bath and stored at −20 °C; GC‐FID and ^1^H NMR spectra afforded a purity >96 %, with traces of dimers. A higher purity could be obtained upon condensation of MCPD vapor under reduced pressure in liquid nitrogen. MCPD was used freshly (within 3 h after distillation).

### Dimerization of MCPD in the Presence of CB7

Freshly distilled MCPD (1.3 μL, ~6 mM, mixture of 1‐ and 2‐MCPD isomers) was added to a solution of CB7 (2 mL, 3 mM, pH 2.8–3.5) in Millipore water. The reaction mixture was allowed to stir at room temperature for 17 h. The products were extracted with dichloromethane (1.5 mL), subsequently dried over anhydrous sodium sulfate, and filtered for analysis. Where required, the pH was adjusted with HCl or NaOH.

### Quantum Chemical Calculations

Density functional theory (DFT) calculations were performed by using the Gaussian 16 program package.^[76]^ Geometry optimization and energy calculations (in the gas phase) were run with the B3LYP functional^[77]^ and Grimme's D3BJ dispersion correction method^[78]^ with the 6‐31+G* basis set. Thermodynamic data of the *endo* transition states for the dimerization of free MCPD are corrected for the Ben‐Naim reference state.[^66^, ^79^]

## Conflict of Interests

The authors declare no conflict of interest.

1

## Supporting information

As a service to our authors and readers, this journal provides supporting information supplied by the authors. Such materials are peer reviewed and may be re‐organized for online delivery, but are not copy‐edited or typeset. Technical support issues arising from supporting information (other than missing files) should be addressed to the authors.

Supporting Information

## Data Availability

The data that support the findings of this study are available in the supplementary material of this article.
