# Supplementary material for: Regioselective Dimerization of Methylcyclopentadiene inside Cucurbit[7]uril
Source: Chemistry. 2025 Feb 5;31(12):e202403964. doi: 10.1002/chem.202403964 (PMC11855239; doi:10.1002/chem.202403964)
Supplement: Supplementary file 1 — Supporting Information [file CHEM-31-e202403964-s001.pdf]

Chemistry–A European Journal

Supporting Information

Regioselective Dimerization of Methylcyclopentadiene inside Cucurbit[7]uril

Khaleel I. Assaf,* Foad N. Tehrani, Guillermo E. Quintero, Robert Hein, Margarita E. Aliaga, and Werner M. Nau*

Supporting Information (SI)

Regioselective Dimerization of Methylcyclopentadiene inside Cucurbit[7]uril

Khaleel I. Assaf,*[a] Foad N. Tehrani,[b] Guillermo E. Quintero,[b,c] Robert Hein,[b] Margarita E. Aliaga,[c] Werner M. Nau*[b]

[a] K. I. Assaf
Department of Chemistry, Faculty of Science
Al-Balqa Applied University
19117 Al-Salt, Jordan
E-mail: khaleel.assaf@bau.edu.jo

[b] F. N. Tehrani, G. E. Quintero, R. Hein, W. M. Nau
School of Science
Constructor University
Campus Ring 1, 28759 Bremen, Germany
E-mail: wnau@constructor.university

[c] G. E. Quintero, M. E. Aliaga
Facultad de Química y de Farmacia, Escuela de Química
Pontificia Universidad Católica de Chile
Casilla 306, Santiago 6094411, Chile

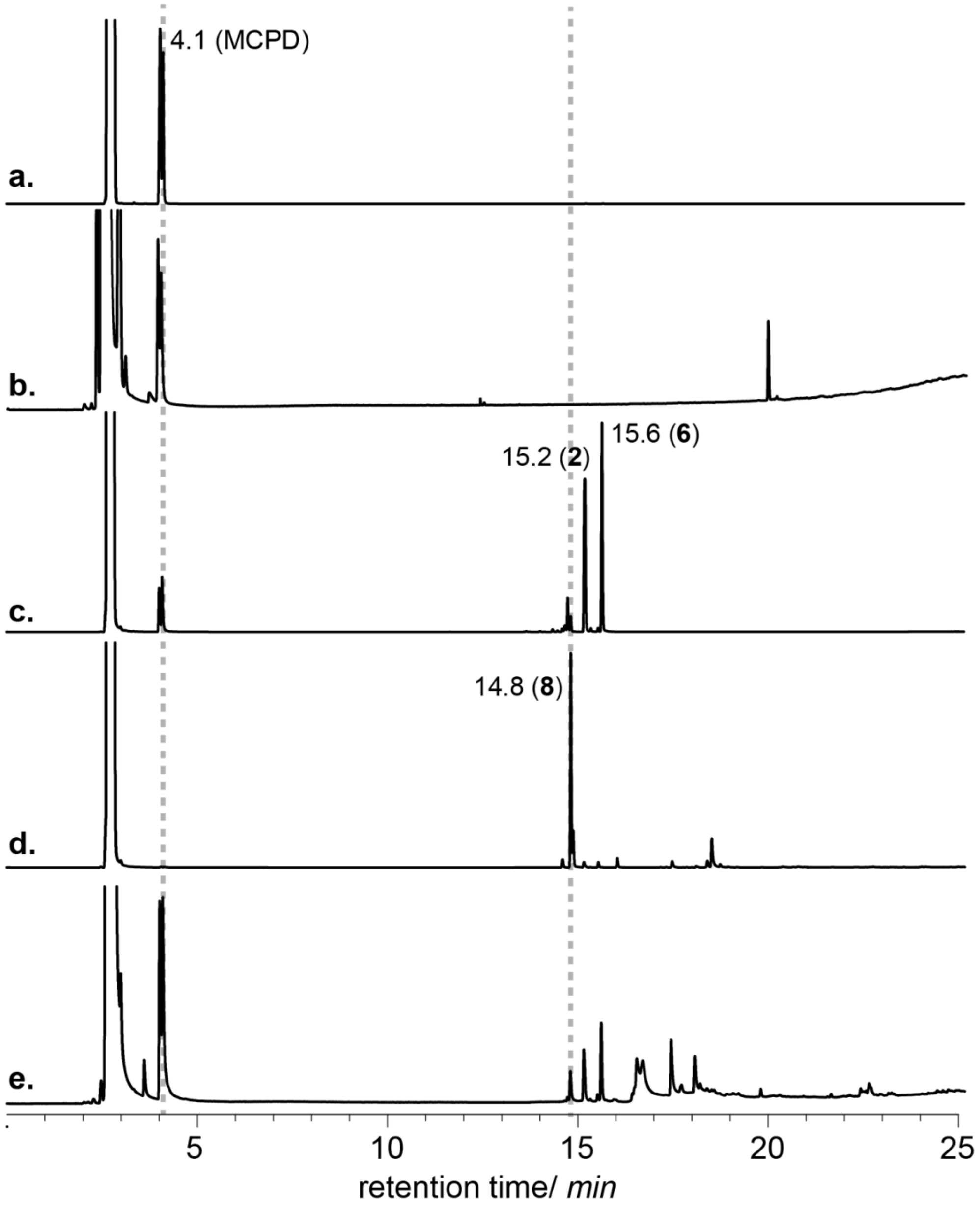

Figure S1. GC-FID chromatogram for **a.** the MCPD monomers and the dimerization products in **b.** aqueous solution (6 mM, pH 3), **c.** the neat liquid, as well as inside the macrocyclic cavities of **d.** CB7 and **e.** β-CD (3 mM host and 6 mM MCPD, pH 2.8), all after 17 h. Note the much faster conversion (disappearance of the MCPD reactant peak) in the presence of CB7.

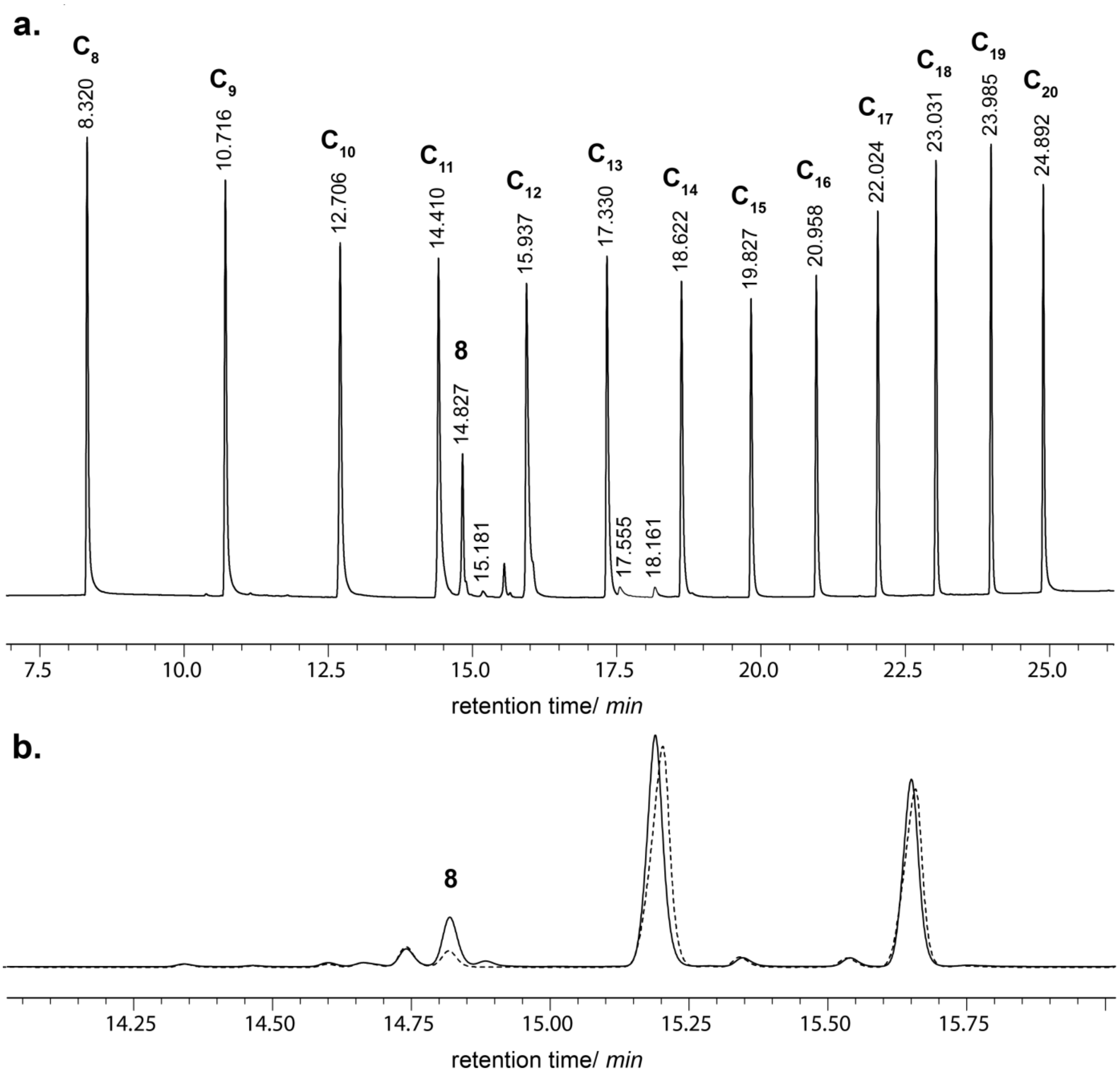

Figure S2 a. GC-FID chromatogram of a standard *n*-alkane mixture with CB7-mediated dimerization of MCPD; data were used to determine retention indices of the dimerization products. **b.** Superimposed GC-FID chromatograms ("co-injection experiment") of neat commercial MCPD dimer (dashed line) and of a mixture of the CB7-mediated cycloaddition products of MCPD with the commercial MCPD dimer (solid lines).

Table S1. Retention indices of MCPD dimers as determined by GC-FID.

	I_T[a]			
dimer	This work[b]	Reported[c]	Reported[d]	Reported[e]
**4**	1113	1104 (9)	1110 (4)	1130 (–17)
**5**	1117	-	-	-
**1**	1122	1105 (17)	1104 (18)	1135 (–13)
**8**	1127	1109 (18)	1109 (18)	1142 (–15)
**2**	1150	1113 (38)	1133 (17)	1162 (–12)
**3**	1162	1134 (28)	1143 (19)	1173 (–11)
**7**	1174	1154 (20)	1155 (19)	1187 (–13)
**6**	1181	1161 (20)	1161 (20)	1193 (–12)
**Target**	1127	-	-	-

[a] Linear retention indices (I_T) are calculated according to the following equation: $I_T = 100 \times \left(n + \frac{t_i - t_n}{t_{n+1} - t_n}\right)$, where t_i is the retention time of the target compound, t_n and t_{n+1} are the retention times of the preceding and succeeding bracketing *n*-alkane, and *n* represents the number of carbon atoms in the preceding bracketing *n*-alkane. [b] Measured in this study by using a VF-1ms GC-column. [c] Taken from ref. [1] by using a HP-PONA GC-column. [d] Taken from ref. [2] by using a HP1 GC-column. [e] Taken from ref. [2, 3] by using a CP-SIL 19 CB GC-column. Values in parentheses represent the deviation from the values measured in this work ($I_{T\ measured} – I_{T\ reported}$).

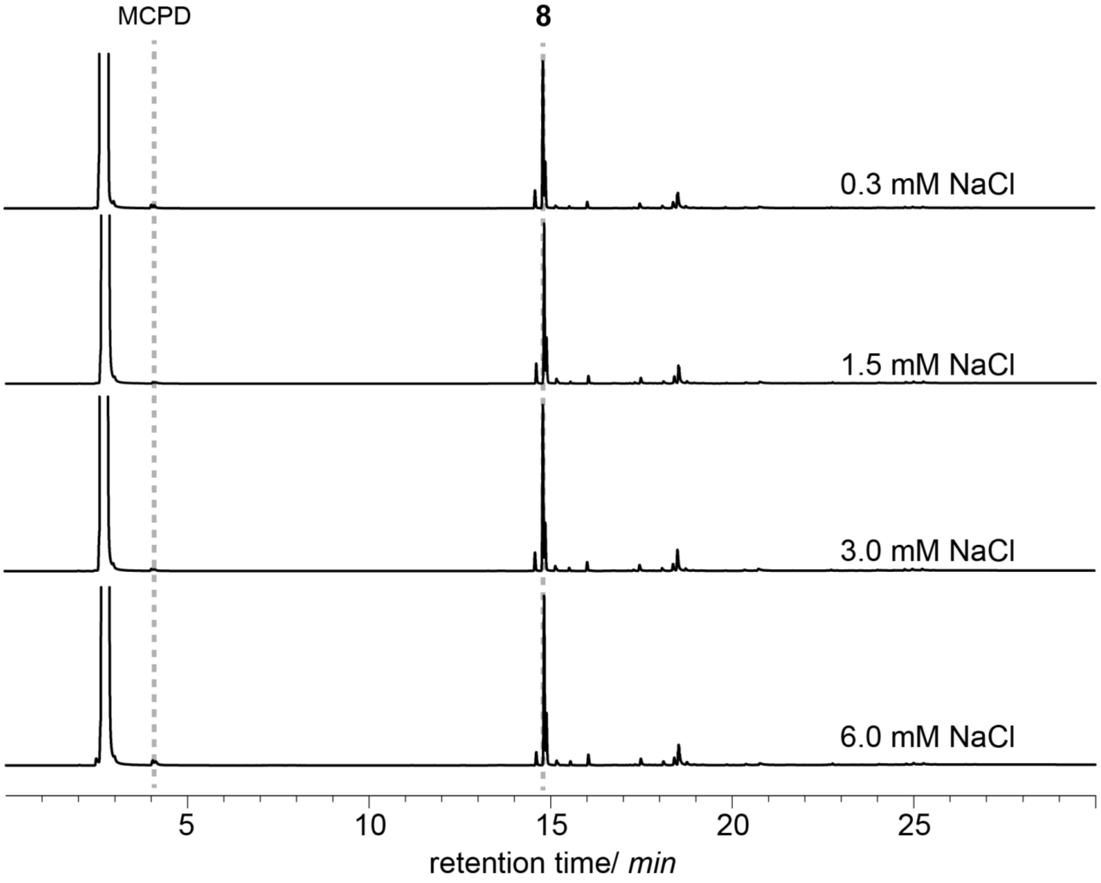

Figure S3. GC-FID chromatograms for the dimerization of MCPD (6 mM) inside CB7 (3 mM) after 15 h at different NaCl concentrations.

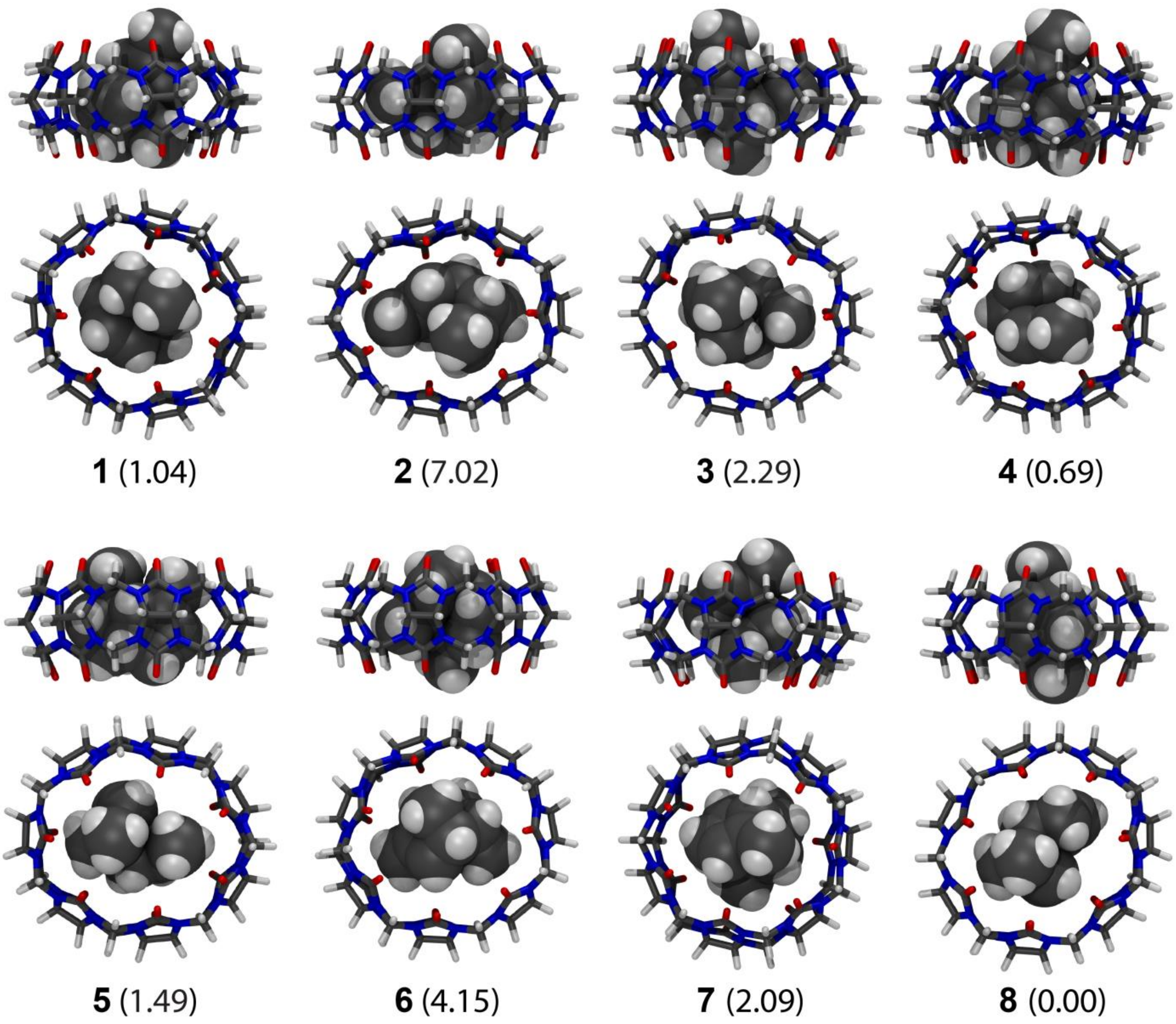

Figure S4. Top and side representations of the optimized structures (at B3LYP-D3(BJ)/6-31+G* of host-guest inclusion complexes of MCPD dimers and CB7. Calculated relative binding energies are given below the structures, in kcal/mol.

Table S2. DFT-Calculated (B3LYP-D3(BJ)/6-31+G*) gas-phase thermodynamic data of the *endo* transition states for the dimerization of free MCPD in kcal/mol. Values are corrected for the Ben-Naim reference state.

Transition state	$\Delta H^{\ddagger}$	$T\Delta S^{\ddagger}$	$\Delta G^{\ddagger}$
**1**	14.6	−9.1	23.6
**2**	14.2	−9.1	23.3
**3**	16.3	−9.4	25.7
**4**	16.2	−9.5	25.7
**5**	14.2	−9.1	23.3
**6**	14.0	−9.4	23.3
**7**	16.7	−9.1	25.8
**8**	15.8	−9.5	25.3

Coordinates of the optimized structure of CB7•dimer inclusion complex

CB7•**1** E(RB3LYP) = -4679.73522409

N	2.406640000	-4.496813000	1.083552000
C	2.315338000	-5.225707000	-0.168809000
C	0.796471000	-5.579239000	-0.266301000
N	0.282748000	-5.212320000	1.028219000
C	1.241536000	-4.622417000	1.840440000
N	0.342128000	-4.787211000	-1.391459000
C	1.398492000	-4.213549000	-2.091199000
N	2.557461000	-4.480713000	-1.375157000
C	-0.918632000	-5.020080000	-2.053751000
N	-2.049511000	-4.401665000	-1.410858000
C	-2.851948000	-3.502719000	-2.104306000
N	-4.020607000	-3.347250000	-1.371528000
C	-4.057597000	-4.170632000	-0.189975000
C	-2.729489000	-4.990744000	-0.278082000
N	-3.944578000	-3.495402000	1.085132000
C	-2.877756000	-3.974033000	1.843150000
N	-2.121424000	-4.793679000	1.014194000
C	-5.114105000	-2.538797000	-1.845003000
N	-5.230018000	-1.250788000	-1.200722000
C	-5.153908000	-0.079208000	-1.949643000
N	-5.541135000	0.963768000	-1.116201000
C	-6.003785000	0.507472000	0.170843000
C	-5.925057000	-1.050961000	0.055269000
N	-5.160083000	0.792766000	1.312674000
C	-4.816115000	-0.355710000	2.015071000
N	-5.232802000	-1.441421000	1.257490000
C	-5.603267000	2.328926000	-1.583616000
N	-4.540267000	3.189042000	-1.121511000
C	-3.458942000	3.529617000	-1.926811000
N	-2.622767000	4.346253000	-1.168679000
C	-3.269913000	4.785864000	0.055007000
C	-4.491890000	3.821444000	0.170819000
N	-2.552312000	4.564909000	1.281988000
C	-3.069530000	3.511414000	2.025766000
N	-4.137219000	2.984959000	1.301291000
C	-1.581719000	5.112514000	-1.818878000
N	-0.269791000	4.945239000	-1.249091000
C	0.763739000	4.354882000	-1.969019000
N	1.941092000	4.599110000	-1.272248000
C	1.746251000	5.443858000	-0.120179000
C	0.199533000	5.676169000	-0.097950000

N	2.015981000	4.856246000	1.172192000
C	0.883753000	4.775624000	1.974187000
N	-0.198128000	5.167666000	1.195167000
C	3.230025000	4.330735000	-1.853923000
N	3.975389000	3.271100000	-1.212039000
C	4.428275000	2.190919000	-1.961951000
N	5.380343000	1.528418000	-1.193276000
C	5.580969000	2.139729000	0.093869000
C	4.773554000	3.472298000	-0.016036000
N	4.970717000	1.485348000	1.238799000
C	4.164113000	2.357410000	1.966212000
N	4.062690000	3.532587000	1.233341000
C	6.009201000	0.312490000	-1.645319000
N	5.430285000	-0.909767000	-1.138077000
C	5.759503000	-1.507390000	0.131886000
C	5.109759000	-2.927978000	0.048197000
N	4.378184000	-2.884515000	-1.199555000
C	4.675828000	-1.749045000	-1.948942000
N	4.331469000	-3.015278000	1.259560000
C	4.409891000	-1.863238000	2.033083000
N	5.140974000	-0.931781000	1.305079000
O	3.966633000	-1.715847000	3.154221000
C	5.543216000	0.324537000	1.884040000
C	3.671562000	-4.208211000	1.727947000
C	3.852536000	-4.064835000	-1.844383000
O	4.377110000	-1.553213000	-3.108748000
O	4.100319000	1.908347000	-3.096091000
O	3.680753000	2.148787000	3.060886000
C	3.339027000	4.681904000	1.719929000
O	0.663191000	3.792748000	-3.040845000
O	0.853430000	4.469044000	3.149519000
C	-1.494261000	5.406938000	1.781238000
O	-3.301880000	3.214807000	-3.089034000
O	-2.695262000	3.153357000	3.123699000
C	-5.073639000	2.089476000	1.936767000
O	-4.854189000	0.009755000	-3.122542000
O	-4.295468000	-0.402190000	3.112023000
C	-5.045795000	-2.799026000	1.708011000
O	-2.601740000	-2.991288000	-3.177706000
O	-2.683013000	-3.766049000	3.023278000
C	-1.023134000	-5.579906000	1.522210000
O	1.329741000	-3.635433000	-3.157343000
O	1.106204000	-4.318916000	3.009311000
H	-1.092859000	-6.107508000	-2.122415000

H	-0.842027000	-4.592858000	-3.056267000
H	-6.050332000	-3.101619000	-1.709869000
H	-4.944693000	-2.347105000	-2.907159000
H	-5.536635000	2.294724000	-2.673418000
H	-6.564587000	2.764718000	-1.278928000
H	-1.537905000	4.771060000	-2.855540000
H	-1.845872000	6.182941000	-1.789475000
H	3.827059000	5.257475000	-1.833037000
H	3.064740000	4.019389000	-2.887972000
H	5.906597000	0.285716000	-2.732419000
H	7.071486000	0.343830000	-1.367573000
H	3.753470000	-3.829878000	-2.906583000
H	4.557617000	-4.898577000	-1.707423000
H	2.970514000	-6.109683000	-0.134850000
H	0.606916000	-6.646879000	-0.457859000
H	-2.890885000	-6.065700000	-0.456283000
H	-4.962008000	-4.798680000	-0.201468000
H	-6.912408000	-1.536569000	0.018163000
H	-7.022884000	0.880922000	0.355614000
H	-5.442299000	4.340021000	0.370706000
H	-3.554869000	5.846129000	-0.029105000
H	-0.084931000	6.736634000	-0.185748000
H	2.327402000	6.372526000	-0.234433000
H	5.410079000	4.362862000	-0.130914000
H	6.655488000	2.291146000	0.276465000
H	6.853061000	-1.533709000	0.259102000
H	5.848320000	-3.744350000	0.019098000
H	-1.006621000	-5.443846000	2.605854000
H	-1.202581000	-6.638282000	1.279396000
H	-5.977363000	-3.357138000	1.530709000
H	-4.833371000	-2.759874000	2.778902000
H	-6.072921000	2.555153000	1.944616000
H	-4.728704000	1.933039000	2.961770000
H	-1.779265000	6.456819000	1.613932000
H	-1.398917000	5.212210000	2.851865000
H	3.938739000	5.579140000	1.508281000
H	3.219534000	4.558655000	2.798673000
H	6.642173000	0.403143000	1.854702000
H	5.198144000	0.325122000	2.920871000
H	3.465055000	-4.067041000	2.791413000
H	4.349346000	-5.065146000	1.589521000
C	-0.150262000	1.472704000	-0.279250000
C	-0.310792000	0.319956000	-1.264545000
C	-0.248817000	-1.011776000	-0.407678000

C	1.110975000	1.469215000	0.174656000
C	1.216950000	-1.012751000	0.153055000
C	1.819645000	0.291598000	-0.478847000
C	-1.110722000	-1.078986000	0.821094000
C	1.086374000	-1.044534000	1.692569000
C	-0.403569000	-1.063440000	1.961964000
C	1.122835000	0.280820000	-1.859947000
H	-0.964965000	2.101811000	0.066912000
C	-1.492101000	0.402502000	-2.210946000
H	-0.432496000	-1.846179000	-1.098266000
H	1.515981000	2.081386000	0.971942000
H	1.781250000	-1.875000000	-0.216642000
H	2.911171000	0.320107000	-0.462823000
H	-2.197885000	-1.091543000	0.776081000
H	1.557545000	-0.177179000	2.174794000
H	1.561575000	-1.919911000	2.144764000
C	-0.909921000	-1.048819000	3.368542000
H	1.352738000	1.159719000	-2.468340000
H	1.321157000	-0.628789000	-2.439133000
H	-2.432629000	0.446333000	-1.646210000
H	-1.545426000	-0.475063000	-2.864275000
H	-1.444672000	1.297347000	-2.839334000
H	-0.544472000	-1.928990000	3.913436000
H	-2.000102000	-1.038581000	3.416161000
H	-0.530519000	-0.161028000	3.893856000

CB7•**2** E(RB3LYP) = -4679.72881826

N	-0.133870000	-4.592142000	-1.260827000
C	0.305813000	-5.325666000	-0.094956000
C	1.851545000	-5.094474000	-0.073865000
N	2.092697000	-4.313843000	-1.260915000
C	0.932695000	-4.064363000	-1.985967000
N	2.078044000	-4.433672000	1.191301000
C	0.923719000	-4.350819000	1.961598000
N	-0.130730000	-4.815761000	1.181325000
C	3.388023000	-4.272964000	1.779043000
N	4.190740000	-3.206380000	1.225044000
C	4.572937000	-2.113412000	1.992925000
N	5.533948000	-1.412867000	1.272072000
C	5.923469000	-2.088529000	0.053121000

C	4.955597000	-3.322894000	0.006370000
N	5.662034000	-1.419428000	-1.200138000
C	4.707272000	-2.069980000	-1.976369000
N	4.224067000	-3.134246000	-1.227519000
C	6.144613000	-0.209050000	1.791448000
N	5.640148000	1.032862000	1.244838000
C	4.795616000	1.859955000	1.977026000
N	4.556256000	2.993860000	1.207394000
C	5.290803000	2.991034000	-0.035491000
C	6.120319000	1.659716000	0.030666000
N	4.512378000	2.839857000	-1.248864000
C	4.892785000	1.725879000	-1.988385000
N	5.809860000	1.015475000	-1.223347000
C	3.803271000	4.113559000	1.720865000
N	2.489990000	4.297374000	1.135179000
C	1.344346000	4.332263000	1.928693000
N	0.299549000	4.795932000	1.133777000
C	0.760113000	5.288187000	-0.143586000
C	2.288829000	4.968195000	-0.130276000
N	0.274584000	4.594403000	-1.315320000
C	1.308253000	4.018422000	-2.052308000
N	2.478043000	4.169157000	-1.313442000
C	-0.979485000	5.170394000	1.700186000
N	-2.116843000	4.472108000	1.151979000
C	-2.933237000	3.680708000	1.956223000
N	-4.057516000	3.353326000	1.207473000
C	-4.120988000	4.071647000	-0.040738000
C	-2.748037000	4.819416000	-0.100166000
N	-4.133816000	3.278910000	-1.246092000
C	-3.011470000	3.496780000	-2.036319000
N	-2.143012000	4.306437000	-1.309939000
C	-5.183961000	2.658381000	1.781876000
N	-5.417506000	1.332395000	1.257708000
C	-5.041890000	0.204422000	1.974822000
N	-5.486270000	-0.903218000	1.266043000
C	-6.257770000	-0.552264000	0.093513000
C	-6.180793000	1.018774000	0.069025000
N	-5.716245000	-0.935137000	-1.191455000
C	-5.336832000	0.154919000	-1.965878000
N	-5.562771000	1.298216000	-1.210196000
C	-5.381363000	-2.231105000	1.819783000
N	-4.339316000	-3.046898000	1.242544000
C	-4.499258000	-3.808851000	0.023866000
C	-3.176530000	-4.642610000	-0.049270000

N	-2.524788000	-4.355555000	1.206194000
C	-3.243093000	-3.460124000	1.989846000
N	-2.530640000	-4.137992000	-1.244127000
C	-3.366500000	-3.301002000	-1.980617000
N	-4.502426000	-3.067774000	-1.212096000
O	-3.159533000	-2.885089000	-3.102484000
C	-5.620483000	-2.285462000	-1.697197000
C	-1.419538000	-4.809034000	-1.883248000
C	-1.423653000	-5.115595000	1.749225000
O	-2.983198000	-3.135790000	3.131699000
O	-4.475795000	0.191804000	3.049566000
O	-4.933865000	0.117639000	-3.110618000
C	-5.313268000	2.613023000	-1.753095000
O	-2.732304000	3.391276000	3.118354000
O	-2.840009000	3.096296000	-3.170813000
C	-0.993314000	4.909030000	-1.938690000
O	1.278214000	4.045073000	3.107684000
O	1.212559000	3.515310000	-3.153370000
C	3.778895000	3.910529000	-1.881798000
O	4.379314000	1.654843000	3.099101000
O	4.530165000	1.452578000	-3.114021000
C	6.351216000	-0.244162000	-1.679706000
O	4.173555000	-1.844048000	3.108482000
O	4.391549000	-1.786374000	-3.113807000
C	3.401999000	-4.150305000	-1.842483000
O	0.853890000	-3.987180000	3.119416000
O	0.866359000	-3.535547000	-3.077733000
H	3.943132000	-5.219287000	1.673404000
H	3.237201000	-4.047220000	2.837088000
H	7.228337000	-0.259140000	1.616793000
H	5.940635000	-0.188430000	2.864585000
H	3.653591000	3.939037000	2.788761000
H	4.392655000	5.031604000	1.569963000
H	-0.940240000	4.940168000	2.767091000
H	-1.123965000	6.254034000	1.557130000
H	-6.093718000	3.258020000	1.630793000
H	-4.979355000	2.552707000	2.849797000
H	-5.146512000	-2.122634000	2.881364000
H	-6.348348000	-2.742045000	1.699277000
H	-1.368801000	-4.884658000	2.815420000
H	-1.632991000	-6.187901000	1.607175000
H	0.023329000	-6.386161000	-0.191961000
H	2.434330000	-6.028479000	-0.117229000
H	5.485218000	-4.288143000	-0.013905000

H	6.985797000	-2.369213000	0.107404000
H	7.205247000	1.826786000	0.102406000
H	5.920381000	3.891386000	-0.102814000
H	2.922897000	5.868037000	-0.180374000
H	0.540635000	6.364482000	-0.230392000
H	-2.851270000	5.914205000	-0.172230000
H	-4.987320000	4.752472000	-0.036582000
H	-7.166734000	1.503213000	0.121679000
H	-7.284259000	-0.934620000	0.194776000
H	-5.405369000	-4.431090000	0.087561000
H	-3.348021000	-5.726371000	-0.143704000
H	3.263150000	-3.855829000	-2.885229000
H	3.929953000	-5.116708000	-1.793017000
H	7.405823000	-0.304004000	-1.382876000
H	6.267742000	-0.249069000	-2.768772000
H	4.375739000	4.835666000	-1.832513000
H	3.627397000	3.619883000	-2.924009000
H	-1.119422000	6.005013000	-1.945043000
H	-0.954030000	4.534968000	-2.964115000
H	-6.187806000	3.250897000	-1.558514000
H	-5.174666000	2.486885000	-2.829449000
H	-6.547906000	-2.822213000	-1.455239000
H	-5.506976000	-2.204748000	-2.780663000
H	-1.357149000	-4.423282000	-2.903161000
H	-1.622951000	-5.893297000	-1.905560000
C	-1.097789000	0.793118000	1.178996000
C	-1.741816000	-0.540685000	0.833206000
C	-0.649184000	-1.390367000	0.058216000
C	-0.948688000	1.457899000	0.018583000
C	-0.528956000	-0.629030000	-1.314526000
C	-1.495068000	0.583589000	-1.106056000
C	0.722807000	-1.278498000	0.650929000
C	0.954406000	-0.247413000	-1.469875000
C	1.543034000	-0.544324000	-0.111240000
C	-2.633443000	-0.122440000	-0.347207000
C	-0.603103000	1.105767000	2.550704000
H	-1.011196000	-2.415173000	-0.049451000
H	-0.418621000	2.391831000	-0.120293000
H	-0.888815000	-1.232724000	-2.152005000
H	0.953796000	-1.590560000	1.666357000
H	1.087460000	0.804646000	-1.757590000
H	1.455278000	-0.838938000	-2.248511000
C	2.827263000	0.067998000	0.314573000
H	-3.413580000	0.575461000	-0.051860000

H	-3.060223000	-0.962240000	-0.896096000
H	3.664785000	-0.231229000	-0.318068000
H	3.054694000	-0.165313000	1.355502000
H	2.743386000	1.158932000	0.225956000
H	-2.202100000	-1.058923000	1.674865000
H	-1.756040000	1.088357000	-2.036897000
H	-0.155078000	2.098442000	2.620922000
H	0.155969000	0.372456000	2.853926000
H	-1.419228000	1.046880000	3.280199000

CB7•**3** E(RB3LYP) = -4679.73507615

N	-3.215786000	4.208868000	1.166786000
C	-3.003475000	4.889601000	-0.084846000
C	-1.532469000	5.404274000	0.021066000
N	-1.040652000	4.750465000	1.219246000
C	-2.076662000	4.190646000	1.963116000
N	-0.941253000	4.997821000	-1.227566000
C	-1.808546000	4.247325000	-2.012355000
N	-2.976255000	4.070774000	-1.277505000
C	0.303658000	5.510397000	-1.745920000
N	1.488030000	4.913758000	-1.180233000
C	2.394352000	4.203223000	-1.962021000
N	3.502554000	3.944045000	-1.162616000
C	3.450586000	4.637993000	0.102762000
C	2.014963000	5.254216000	0.117512000
N	3.481352000	3.819024000	1.288630000
C	2.289463000	3.867013000	2.005608000
N	1.385276000	4.619716000	1.256939000
C	4.711089000	3.375476000	-1.706303000
N	5.066207000	2.086769000	-1.162498000
C	4.965900000	0.937079000	-1.935762000
N	5.580308000	-0.088817000	-1.228181000
C	6.188895000	0.357124000	0.004097000
C	5.842687000	1.888833000	0.041132000
N	5.623443000	-0.142228000	1.238078000
C	5.066346000	0.858721000	2.023152000
N	5.149857000	2.041330000	1.301066000
C	5.697352000	-1.413192000	-1.784989000
N	4.789016000	-2.389326000	-1.226902000
C	3.773658000	-2.956584000	-1.991721000
N	3.239554000	-4.004097000	-1.249429000
C	3.948339000	-4.235958000	-0.014920000
C	5.073669000	-3.149490000	-0.030450000
N	3.222180000	-3.955600000	1.205036000

C	3.839815000	-2.972108000	1.974666000
N	4.896899000	-2.468220000	1.227170000
C	2.271979000	-4.914062000	-1.809318000
N	0.962069000	-4.859316000	-1.199836000
C	-0.164187000	-4.568168000	-1.964568000
N	-1.278311000	-4.901965000	-1.203068000
C	-0.934497000	-5.548233000	0.039618000
C	0.630321000	-5.559031000	0.021907000
N	-1.250550000	-4.836032000	1.258000000
C	-0.122216000	-4.563830000	2.023372000
N	0.985756000	-4.903933000	1.257229000
C	-2.607344000	-4.891780000	-1.765888000
N	-3.486434000	-3.881554000	-1.231732000
C	-3.933968000	-2.821259000	-2.010313000
N	-4.887243000	-2.139461000	-1.263595000
C	-5.200758000	-2.799684000	-0.017552000
C	-4.197254000	-4.000362000	0.018345000
N	-4.903397000	-2.077322000	1.195440000
C	-3.918123000	-2.687955000	1.959602000
N	-3.432887000	-3.765417000	1.222708000
C	-5.693821000	-1.092874000	-1.844726000
N	-5.436318000	0.230036000	-1.333950000
C	-5.982076000	0.773198000	-0.115156000
C	-5.539270000	2.275286000	-0.160270000
N	-4.648537000	2.322114000	-1.302139000
C	-4.675290000	1.147664000	-2.044599000
N	-4.951835000	2.489472000	1.138981000
C	-4.924295000	1.335919000	1.916182000
N	-5.417292000	0.300431000	1.131854000
O	-4.590985000	1.265802000	3.081569000
C	-5.702329000	-0.991497000	1.709852000
C	-4.504218000	3.762957000	1.649880000
C	-4.168852000	3.546006000	-1.895123000
O	-4.172961000	0.974284000	-3.136892000
O	-3.591981000	-2.570341000	-3.148415000
O	-3.579018000	-2.376083000	3.083603000
C	-2.576478000	-4.760374000	1.824472000
O	-0.172151000	-4.146291000	-3.103151000
O	-0.107954000	-4.149485000	3.165507000
C	2.315368000	-4.902470000	1.813366000
O	3.446422000	-2.629944000	-3.114086000
O	3.540939000	-2.652348000	3.107325000
C	5.805886000	-1.477775000	1.752770000
O	4.484167000	0.855397000	-3.046841000
O	4.636495000	0.733377000	3.152099000
C	4.699215000	3.291421000	1.861138000
O	2.262391000	3.913509000	-3.133261000
O	2.089379000	3.383302000	3.100916000
C	0.187450000	5.142828000	1.872064000
O	-1.602922000	3.861620000	-3.145613000
O	-2.011164000	3.803199000	3.112692000

H	0.332916000	6.597864000	-1.575560000
H	0.320705000	5.299984000	-2.817713000
H	5.547237000	4.072274000	-1.538919000
H	4.544540000	3.242452000	-2.777960000
H	5.466769000	-1.332983000	-2.850013000
H	6.729961000	-1.767077000	-1.648552000
H	2.149013000	-4.646665000	-2.861392000
H	2.661346000	-5.941485000	-1.726126000
H	-3.064554000	-5.880806000	-1.609420000
H	-2.503343000	-4.692964000	-2.835070000
H	-5.469568000	-1.074801000	-2.913788000
H	-6.756057000	-1.329250000	-1.686901000
H	-3.922916000	3.328978000	-2.937387000
H	-4.966383000	4.305404000	-1.848199000
H	-3.743256000	5.697268000	-0.200563000
H	-1.458599000	6.497564000	0.132804000
H	2.010672000	6.347806000	0.250070000
H	4.251443000	5.392768000	0.150803000
H	6.732008000	2.536662000	0.022134000
H	7.270302000	0.155129000	-0.018478000
H	6.089102000	-3.570982000	-0.088516000
H	4.336432000	-5.266251000	0.008727000
H	1.058831000	-6.573576000	-0.001333000
H	-1.378543000	-6.555672000	0.070820000
H	-4.692345000	-4.982293000	0.075735000
H	-6.256656000	-3.111823000	-0.016866000
H	-7.074840000	0.640508000	-0.105263000
H	-6.377357000	2.972655000	-0.312155000
H	0.146134000	4.756067000	2.892718000
H	0.250692000	6.244659000	1.893813000
H	5.493498000	4.043476000	1.745394000
H	4.508324000	3.109016000	2.921260000
H	6.836082000	-1.802131000	1.547143000
H	5.636722000	-1.429454000	2.831030000
H	2.742501000	-5.914495000	1.722210000
H	2.222545000	-4.631591000	2.867618000
H	-3.060569000	-5.746298000	1.737574000
H	-2.460201000	-4.495703000	2.878070000
H	-6.765225000	-1.232169000	1.552932000
H	-5.492005000	-0.913383000	2.779118000
H	-4.421538000	3.657362000	2.733974000
H	-5.253473000	4.527951000	1.402646000
C	0.392763000	1.190968000	-0.960786000
C	-0.807390000	0.637127000	-1.705126000
C	-1.716369000	-0.086493000	-0.634882000
C	1.146555000	0.153864000	-0.560556000
C	-0.843425000	-1.324897000	-0.213886000
C	0.458197000	-1.114685000	-1.059931000
C	-1.957078000	0.634315000	0.661384000
C	-0.713109000	-1.258513000	1.321472000
C	-1.395753000	0.032001000	1.720747000

C	-0.163480000	-0.600133000	-2.379325000
H	-2.646338000	-0.390495000	-1.128711000
C	2.365964000	0.168138000	0.299341000
H	-1.313492000	-2.260681000	-0.527956000
H	-2.484371000	1.582186000	0.706723000
H	0.328049000	-1.280469000	1.667443000
H	-1.199796000	-2.101317000	1.821531000
C	-1.380457000	0.471628000	3.149387000
H	0.590094000	-0.334109000	-3.125983000
H	-0.893620000	-1.290372000	-2.815928000
H	-1.846597000	-0.294594000	3.782389000
H	-1.908345000	1.413749000	3.301351000
H	-0.344882000	0.593370000	3.497212000
H	-1.343679000	1.328405000	-2.356104000
H	1.096428000	-2.000597000	-1.116273000
H	2.730423000	1.190098000	0.436604000
H	3.168169000	-0.428492000	-0.148962000
H	2.179982000	-0.253006000	1.295893000
H	0.534446000	2.232001000	-0.684980000

CB7•**4** E(RB3LYP) = -4679.73578856

N	-5.295800000	0.379313000	1.179125000
C	-5.974759000	0.861641000	-0.012604000
C	-5.493179000	2.343359000	-0.113848000
N	-4.897576000	2.584431000	1.172750000
C	-4.782653000	1.431306000	1.937792000
N	-4.598949000	2.317836000	-1.257460000
C	-4.750031000	1.153786000	-2.004033000
N	-5.567948000	0.299210000	-1.271902000
C	-4.080392000	3.514332000	-1.881354000
N	-2.908334000	4.063406000	-1.248110000
C	-1.698946000	4.149587000	-1.927881000
N	-0.863936000	4.972526000	-1.179991000
C	-1.499960000	5.479036000	0.007968000
C	-2.964542000	4.942605000	-0.101563000
N	-1.041348000	4.933447000	1.269213000
C	-2.073256000	4.361156000	2.004998000
N	-3.192044000	4.328479000	1.182940000
C	0.397493000	5.445404000	-1.688244000
N	1.562526000	4.851179000	-1.067237000
C	2.575463000	4.312667000	-1.858282000
N	3.672021000	4.099198000	-1.029457000
C	3.464085000	4.594344000	0.306110000
C	2.067765000	5.286297000	0.222603000

N	3.278443000	3.606785000	1.351653000
C	2.099269000	3.818741000	2.064473000
N	1.379897000	4.794038000	1.385849000
C	4.911634000	3.542090000	-1.516380000
N	5.179811000	2.180042000	-1.117437000
C	4.972257000	1.105067000	-1.975423000
N	5.346720000	-0.043414000	-1.285016000
C	6.061038000	0.264485000	-0.060609000
C	5.728531000	1.775374000	0.151265000
N	5.587167000	-0.354956000	1.149466000
C	4.858278000	0.519775000	1.945312000
N	4.817891000	1.742444000	1.281465000
C	5.435873000	-1.313930000	-1.960906000
N	4.572047000	-2.332051000	-1.415602000
C	3.461277000	-2.780932000	-2.119032000
N	2.986670000	-3.907789000	-1.461241000
C	3.811960000	-4.298125000	-0.345399000
C	4.947713000	-3.220125000	-0.339444000
N	3.220725000	-4.177376000	0.971171000
C	3.936649000	-3.315247000	1.792779000
N	4.889395000	-2.686476000	0.999867000
C	1.981030000	-4.750824000	-2.052030000
N	0.717594000	-4.751706000	-1.353870000
C	-0.457437000	-4.485911000	-2.051188000
N	-1.511055000	-4.930710000	-1.263457000
C	-1.073403000	-5.534530000	-0.032656000
C	0.479416000	-5.568392000	-0.179331000
N	-1.269490000	-4.768500000	1.182379000
C	-0.086383000	-4.634446000	1.909047000
N	0.949934000	-5.047533000	1.076600000
C	-2.872382000	-4.923575000	-1.736552000
N	-3.707554000	-3.891550000	-1.177045000
C	-4.305942000	-3.945054000	0.131744000
C	-5.290448000	-2.728782000	0.125499000
N	-4.998540000	-2.064760000	-1.129046000
C	-4.154395000	-2.820185000	-1.939060000
N	-4.947099000	-2.022129000	1.332046000
C	-3.887156000	-2.598308000	2.018714000
N	-3.435940000	-3.672626000	1.259059000
O	-3.465978000	-2.256119000	3.106086000
C	-2.551603000	-4.661162000	1.835892000
C	-5.643068000	-0.865672000	1.832037000
C	-5.845769000	-1.044773000	-1.708720000
O	-3.899364000	-2.612736000	-3.107631000

O	-0.544262000	-4.003621000	-3.162750000
O	0.016302000	-4.288720000	3.067850000
C	2.311983000	-5.153015000	1.533690000
O	3.019591000	-2.306752000	-3.146749000
O	3.791465000	-3.172735000	2.990810000
C	5.820235000	-1.725677000	1.540345000
O	4.587386000	1.165889000	-3.125181000
O	4.390859000	0.275327000	3.038805000
C	4.386812000	2.928005000	1.979630000
O	2.529289000	4.112974000	-3.055149000
O	1.782953000	3.286251000	3.108932000
C	0.170253000	5.365000000	1.922299000
O	-1.439558000	3.656031000	-3.007265000
O	-2.021543000	4.007055000	3.165916000
C	-4.473594000	3.873776000	1.661775000
O	-4.302401000	0.940915000	-3.112352000
O	-4.363609000	1.362372000	3.075138000
H	-4.870140000	4.284137000	-1.898158000
H	-3.803466000	3.246455000	-2.903699000
H	0.434314000	6.540037000	-1.563838000
H	0.445153000	5.192965000	-2.749767000
H	4.855397000	3.552982000	-2.607190000
H	5.743369000	4.174245000	-1.176409000
H	5.133961000	-1.142458000	-2.997014000
H	6.474820000	-1.678325000	-1.928838000
H	2.369520000	-5.781590000	-2.102740000
H	1.789783000	-4.376414000	-3.060239000
H	-2.841298000	-4.755465000	-2.815420000
H	-3.321931000	-5.903489000	-1.520332000
H	-5.681682000	-1.072163000	-2.788324000
H	-6.896496000	-1.275313000	-1.481656000
H	-7.063842000	0.762773000	0.111064000
H	-6.310971000	3.058751000	-0.290344000
H	-3.710337000	5.734545000	-0.272584000
H	-1.436279000	6.578273000	0.028643000
H	2.120882000	6.385850000	0.254856000
H	4.277890000	5.282836000	0.582656000
H	6.609751000	2.389055000	0.392895000
H	7.135808000	0.064692000	-0.188082000
H	5.947984000	-3.637203000	-0.532907000
H	4.184345000	-5.323526000	-0.495293000
H	0.881286000	-6.581359000	-0.340462000
H	-1.526264000	-6.532820000	0.074610000
H	-4.809426000	-4.913560000	0.275793000

H	-6.350513000	-3.024505000	0.152051000
H	-4.396782000	3.795408000	2.748519000
H	-5.237901000	4.615249000	1.390683000
H	0.249703000	6.461925000	1.861486000
H	0.093433000	5.054582000	2.966776000
H	5.234139000	3.628902000	2.069158000
H	4.059641000	2.614698000	2.973892000
H	6.840396000	-2.000319000	1.239881000
H	5.725045000	-1.776773000	2.627265000
H	2.683154000	-6.164238000	1.303536000
H	2.307668000	-4.995978000	2.614689000
H	-3.055465000	-5.642292000	1.816008000
H	-2.360333000	-4.365831000	2.870101000
H	-5.375661000	-0.759155000	2.886000000
H	-6.726079000	-1.036456000	1.734601000
C	-1.460697000	1.112335000	0.313421000
C	-1.803859000	-0.175091000	-0.416909000
C	-0.769553000	-1.268924000	0.067870000
C	-0.300019000	1.556481000	-0.190162000
C	0.583573000	-0.742115000	-0.524222000
C	0.161599000	0.579371000	-1.269023000
C	-0.512353000	-1.352620000	1.542721000
C	1.548369000	-0.583532000	0.674736000
C	0.719283000	-0.953632000	1.889876000
C	-1.218321000	0.126872000	-1.815956000
H	-1.980289000	1.493316000	1.186418000
H	-1.071715000	-2.231248000	-0.366188000
H	0.289607000	2.391858000	0.173582000
H	0.984384000	-1.431294000	-1.274186000
C	1.191402000	1.097870000	-2.253351000
H	-1.269244000	-1.643206000	2.264654000
H	1.940880000	0.440920000	0.761982000
H	2.430721000	-1.233071000	0.584127000
C	1.253460000	-0.818877000	3.280613000
H	-1.740884000	0.924619000	-2.348844000
H	-1.156118000	-0.759275000	-2.458820000
H	2.170776000	-1.400786000	3.419238000
H	0.514205000	-1.153370000	4.015407000
H	1.509097000	0.228376000	3.490898000
H	-2.845800000	-0.497489000	-0.360568000
H	2.106967000	1.403086000	-1.731115000
H	0.817094000	1.965115000	-2.805617000
H	1.476511000	0.325152000	-2.975785000

CB7•**5** E(RB3LYP) = -4679.73411826

N	-0.573334000	4.906770000	-1.135697000
C	-1.060918000	5.331926000	0.153951000
C	-2.579812000	4.966799000	0.113665000
N	-2.729594000	4.307499000	-1.162941000
C	-1.578065000	4.374521000	-1.937341000
N	-2.764157000	4.153205000	1.289121000
C	-1.591561000	3.982426000	2.013757000
N	-0.566125000	4.599205000	1.298493000
C	-4.058465000	3.818392000	1.836027000
N	-4.695005000	2.674292000	1.230996000
C	-4.977225000	1.535541000	1.973694000
N	-5.676218000	0.665519000	1.143119000
C	-6.069334000	1.287097000	-0.102751000
C	-5.411603000	2.709097000	-0.025618000
N	-5.523010000	0.741355000	-1.321939000
C	-4.728757000	1.644507000	-2.016389000
N	-4.621980000	2.784030000	-1.231059000
C	-6.181354000	-0.598654000	1.631636000
N	-5.459232000	-1.763339000	1.172940000
C	-4.514960000	-2.408656000	1.964232000
N	-3.939786000	-3.409936000	1.186475000
C	-4.664779000	-3.616975000	-0.052632000
C	-5.624924000	-2.383119000	-0.118420000
N	-3.904828000	-3.517134000	-1.271943000
C	-4.216890000	-2.383921000	-2.011632000
N	-5.144084000	-1.653518000	-1.272973000
C	-3.115988000	-4.426949000	1.800137000
N	-1.795727000	-4.560500000	1.229887000
C	-0.651576000	-4.327206000	1.987475000
N	0.429419000	-4.829076000	1.270438000
C	0.030499000	-5.511500000	0.063155000
C	-1.523267000	-5.337072000	0.042135000
N	0.456624000	-4.915553000	-1.182377000
C	-0.614322000	-4.509125000	-1.971365000
N	-1.770476000	-4.684466000	-1.220593000
C	1.743270000	-4.952756000	1.855965000
N	2.768872000	-4.148540000	1.231496000
C	3.501787000	-3.239706000	1.988546000
N	4.601502000	-2.860688000	1.230351000
C	4.696487000	-3.573829000	-0.016913000
C	3.475467000	-4.550438000	0.030566000
N	4.471526000	-2.810632000	-1.225126000

C	3.427765000	-3.327034000	-1.986554000
N	2.815580000	-4.316102000	-1.227423000
C	5.601285000	-1.946385000	1.723047000
N	5.537486000	-0.615309000	1.163635000
C	5.165006000	0.469172000	1.948763000
N	5.363223000	1.614864000	1.188853000
C	6.008135000	1.346817000	-0.075984000
C	6.165928000	-0.214746000	-0.078000000
N	5.242379000	1.605209000	-1.274043000
C	5.008817000	0.460174000	-2.024040000
N	5.515080000	-0.612922000	-1.303437000
C	5.143235000	2.928691000	1.743079000
N	3.957022000	3.603428000	1.271339000
C	3.863061000	4.332794000	0.032952000
C	2.450069000	4.996645000	0.112897000
N	1.854587000	4.379668000	1.283701000
C	2.793079000	3.666341000	2.026134000
N	1.849935000	4.671021000	-1.155187000
C	2.659343000	3.855611000	-1.938670000
N	3.791294000	3.554990000	-1.186607000
O	2.439303000	3.509673000	-3.081861000
C	4.938555000	2.915551000	-1.791349000
C	0.687198000	5.335540000	-1.692790000
C	0.685645000	4.921801000	1.940057000
O	2.637132000	3.221842000	3.145793000
O	4.786118000	0.427029000	3.101564000
O	4.506367000	0.413563000	-3.128992000
C	5.473960000	-1.957739000	-1.822012000
O	3.255981000	-2.885289000	3.124610000
O	3.144451000	-3.017344000	-3.126479000
C	1.762826000	-5.140385000	-1.759924000
O	-0.609808000	-3.815203000	3.088777000
O	-0.553397000	-4.120107000	-3.121096000
C	-3.080708000	-4.568416000	-1.813541000
O	-4.271440000	-2.171558000	3.130177000
O	-3.798942000	-2.108314000	-3.117585000
C	-5.856226000	-0.546678000	-1.871825000
O	-4.705944000	1.351406000	3.143281000
O	-4.265481000	1.489435000	-3.128207000
C	-4.012773000	3.986830000	-1.741044000
O	-1.486151000	3.439231000	3.095551000
O	-1.476609000	4.071977000	-3.110012000
H	-4.714858000	4.696670000	1.735388000
H	-3.918072000	3.578904000	2.892635000

H	-7.235839000	-0.699431000	1.340669000
H	-6.094692000	-0.579347000	2.720551000
H	-2.994230000	-4.146216000	2.848814000
H	-3.628763000	-5.400810000	1.731424000
H	1.667481000	-4.624098000	2.894973000
H	2.044913000	-6.012594000	1.819525000
H	6.596297000	-2.370989000	1.524971000
H	5.444666000	-1.847578000	2.799794000
H	5.033141000	2.803776000	2.822764000
H	6.014945000	3.560911000	1.524218000
H	0.657548000	4.498499000	2.946456000
H	0.782510000	6.019875000	2.000534000
H	-0.874311000	6.409102000	0.289965000
H	-3.243361000	5.844633000	0.161125000
H	-6.148169000	3.527645000	-0.012061000
H	-7.167737000	1.319290000	-0.167768000
H	-6.680158000	-2.659480000	-0.266286000
H	-5.196285000	-4.580935000	-0.019198000
H	-2.071791000	-6.291150000	0.085807000
H	0.353028000	-6.563849000	0.103483000
H	3.767002000	-5.609787000	0.109151000
H	5.666781000	-4.091212000	-0.078122000
H	7.215302000	-0.546156000	-0.087072000
H	6.968984000	1.881474000	-0.124396000
H	4.686512000	5.060300000	-0.035311000
H	2.488599000	6.089624000	0.242472000
H	-3.852215000	3.841434000	-2.811825000
H	-4.701767000	4.829856000	-1.575629000
H	-6.937816000	-0.716152000	-1.763969000
H	-5.585422000	-0.521582000	-2.930193000
H	-3.611570000	-5.525865000	-1.696198000
H	-2.933489000	-4.350851000	-2.873877000
H	2.035275000	-6.198106000	-1.617270000
H	1.686166000	-4.916702000	-2.826365000
H	6.464282000	-2.418606000	-1.687846000
H	5.238255000	-1.882718000	-2.886218000
H	5.819024000	3.563597000	-1.662305000
H	4.714887000	2.797230000	-2.854310000
H	0.654839000	5.119359000	-2.762986000
H	0.798363000	6.419811000	-1.532437000
C	0.838570000	-1.395144000	0.133752000
C	1.126819000	-0.056825000	0.805094000
C	-0.028686000	0.922211000	0.334825000
C	1.041221000	-1.259542000	-1.185492000

C	0.223656000	1.091876000	-1.200679000
C	1.474939000	0.177149000	-1.438419000
C	-1.433397000	0.388390000	0.433591000
C	-1.089669000	0.678924000	-1.913550000
C	-1.988318000	0.256090000	-0.779426000
C	2.251290000	0.448961000	-0.130165000
H	0.089649000	1.860313000	0.894131000
H	0.493793000	2.121522000	-1.449503000
C	-2.040988000	-0.004442000	1.741419000
H	-0.935493000	-0.143222000	-2.625592000
H	-1.519090000	1.499789000	-2.499048000
H	3.154620000	-0.158720000	-0.036436000
H	2.496270000	1.504292000	0.025811000
C	1.403261000	-0.063081000	2.298734000
H	1.990602000	0.362548000	-2.381426000
H	0.422505000	-2.253077000	0.647584000
H	0.821979000	-1.977325000	-1.967513000
H	-2.980250000	-0.153108000	-0.941832000
H	-3.090532000	-0.271285000	1.620730000
H	-1.975354000	0.797542000	2.485761000
H	-1.531972000	-0.875368000	2.173568000
H	0.569349000	-0.498333000	2.858839000
H	1.562584000	0.953471000	2.673199000
H	2.293991000	-0.648230000	2.541400000

CB7•**6** E(RB3LYP) = -4679.73325733

N	4.345105000	3.167031000	-1.159221000
C	4.472516000	3.907523000	0.075764000
C	3.188444000	4.804575000	0.095260000
N	2.539944000	4.494418000	-1.155630000
C	3.258068000	3.581830000	-1.921794000
N	2.500426000	4.387254000	1.298841000
C	3.253347000	3.497789000	2.056080000
N	4.371746000	3.162047000	1.307037000
C	1.412222000	5.124738000	1.896331000
N	0.127581000	4.922273000	1.269125000
C	-0.953092000	4.407037000	1.980038000
N	-2.094957000	4.602392000	1.202202000
C	-1.826014000	5.412713000	0.035011000
C	-0.276971000	5.616921000	0.070996000
N	-2.048553000	4.791966000	-1.246386000
C	-0.885496000	4.650644000	-1.992132000
N	0.172255000	5.052104000	-1.181426000

C	-3.417517000	4.489344000	1.783508000
N	-4.236906000	3.436212000	1.238933000
C	-4.593014000	2.328603000	1.997004000
N	-5.419334000	1.538259000	1.206579000
C	-5.688066000	2.123053000	-0.087170000
C	-4.891202000	3.474899000	-0.049690000
N	-5.138907000	1.450939000	-1.243955000
C	-4.249187000	2.242943000	-1.959047000
N	-4.040773000	3.400972000	-1.216749000
C	-6.020419000	0.328690000	1.717426000
N	-5.450352000	-0.911825000	1.238650000
C	-4.561760000	-1.666284000	1.997505000
N	-4.255458000	-2.808375000	1.258873000
C	-5.096664000	-2.939779000	0.084692000
C	-5.786156000	-1.541546000	-0.012726000
N	-4.421373000	-3.070608000	-1.180374000
C	-4.495242000	-1.916015000	-1.951828000
N	-5.196354000	-0.972163000	-1.207312000
C	-3.619828000	-3.939839000	1.892990000
N	-2.370235000	-4.334879000	1.284523000
C	-1.206962000	-4.396646000	2.048803000
N	-0.254412000	-5.071507000	1.296011000
C	-0.791454000	-5.612208000	0.074252000
C	-2.300261000	-5.208858000	0.129883000
N	-0.332898000	-5.021131000	-1.167301000
C	-1.398255000	-4.594420000	-1.958410000
N	-2.535022000	-4.612213000	-1.159360000
C	1.055171000	-5.379249000	1.815770000
N	2.133323000	-4.628733000	1.214243000
C	2.935589000	-3.788215000	1.980896000
N	4.013466000	-3.413983000	1.190599000
C	4.044838000	-4.104221000	-0.075579000
C	2.762863000	-5.001013000	-0.034608000
N	3.854198000	-3.297559000	-1.260537000
C	2.739206000	-3.687094000	-1.994292000
N	2.065727000	-4.644501000	-1.245669000
C	5.112513000	-2.641822000	1.720603000
N	5.237891000	-1.309096000	1.184584000
C	5.880045000	-0.989676000	-0.066012000
C	6.031349000	0.568484000	-0.014254000
N	5.321096000	0.927751000	1.196358000
C	4.966845000	-0.184913000	1.955717000
N	5.463495000	0.997500000	-1.269815000
C	4.935199000	-0.051119000	-2.014253000
N	5.102924000	-1.211361000	-1.266983000
O	4.461826000	0.025567000	-3.129802000
C	4.905664000	-2.511047000	-1.862561000
C	5.400482000	2.362574000	-1.733966000
C	5.367868000	2.241711000	1.795519000
O	4.559255000	-0.177245000	3.098732000
O	2.750751000	-3.481944000	3.142082000

O	2.433667000	-3.297633000	-3.103867000
C	0.934309000	-5.365053000	-1.773627000
O	-1.065873000	-3.980348000	3.180919000
O	-1.351937000	-4.307731000	-3.137366000
C	-3.841404000	-4.291928000	-1.682981000
O	-4.162608000	-1.402783000	3.113202000
O	-4.075012000	-1.779809000	-3.082733000
C	-5.676591000	0.244515000	-1.819027000
O	-4.283984000	2.116798000	3.152110000
O	-3.787000000	1.999019000	-3.054226000
C	-3.354833000	4.531811000	-1.797014000
O	-0.916753000	3.922785000	3.092471000
O	-0.808774000	4.286662000	-3.148818000
C	1.485457000	5.292798000	-1.732217000
O	3.002154000	3.120547000	3.184164000
O	3.009553000	3.250083000	-3.062978000
H	1.651080000	6.200880000	1.872500000
H	1.326860000	4.786456000	2.931480000
H	-3.937031000	5.452148000	1.658788000
H	-3.291048000	4.268962000	2.846104000
H	-5.884610000	0.346360000	2.801081000
H	-7.091368000	0.331300000	1.472029000
H	-3.401486000	-3.654548000	2.924749000
H	-4.314502000	-4.796802000	1.881927000
H	1.245151000	-6.455167000	1.679557000
H	1.048095000	-5.134847000	2.880475000
H	4.942964000	-2.542665000	2.795278000
H	6.050563000	-3.186906000	1.536376000
H	5.187665000	2.114583000	2.865674000
H	6.364895000	2.676431000	1.630757000
H	5.409439000	4.485571000	0.066681000
H	3.411099000	5.881987000	0.145935000
H	0.022258000	6.675399000	0.132225000
H	-2.392406000	6.355125000	0.099823000
H	-5.537864000	4.363025000	-0.116995000
H	-6.772656000	2.259877000	-0.215470000
H	-6.880054000	-1.597992000	-0.122234000
H	-5.806386000	-3.770323000	0.220443000
H	-2.975375000	-6.067649000	0.273285000
H	-0.633225000	-6.702202000	0.047576000
H	2.983683000	-6.079814000	-0.033966000
H	4.978651000	-4.681346000	-0.163940000
H	6.842954000	-1.518605000	-0.139531000
H	7.077253000	0.903875000	0.054818000
H	1.437352000	5.047298000	-2.795574000
H	1.738080000	6.358028000	-1.604191000
H	-3.982914000	5.426932000	-1.671839000
H	-3.213358000	4.320161000	-2.859531000
H	-6.775621000	0.274850000	-1.751302000
H	-5.367643000	0.218892000	-2.866886000
H	-4.528133000	-5.121742000	-1.460976000

H	-3.730274000	-4.177928000	-2.763688000
H	1.116183000	-6.444681000	-1.650161000
H	0.856520000	-5.121400000	-2.835672000
H	5.848836000	-3.078602000	-1.809214000
H	4.629324000	-2.346704000	-2.906767000
H	5.220845000	2.322183000	-2.810731000
H	6.366096000	2.846908000	-1.531148000
C	-1.304076000	0.202032000	-0.446780000
C	-0.799056000	-1.233835000	-0.546487000
C	0.667918000	-1.246903000	0.030646000
C	-0.642493000	0.916042000	-1.371064000
C	1.445759000	-0.370813000	-1.013561000
C	0.337576000	-0.010520000	-2.064719000
C	0.883030000	-0.557640000	1.356443000
C	2.043140000	0.811011000	-0.210752000
C	1.611963000	0.556556000	1.210769000
C	-0.482035000	-1.322311000	-2.059012000
H	1.013644000	-2.289305000	0.055461000
H	2.228637000	-0.943621000	-1.514004000
C	0.303401000	-1.075139000	2.635723000
H	1.668515000	1.776802000	-0.575604000
H	3.131452000	0.858681000	-0.297155000
H	-1.374745000	-1.273708000	-2.688455000
H	0.114732000	-2.195436000	-2.336967000
C	-2.235576000	0.698352000	0.602889000
H	-0.700858000	1.988208000	-1.520911000
H	1.861345000	1.217482000	2.035857000
H	0.471189000	-0.370488000	3.456647000
H	0.748926000	-2.033403000	2.922396000
H	-0.776589000	-1.247836000	2.550514000
H	-1.455683000	-1.996028000	-0.116762000
H	0.733697000	0.328862000	-3.023581000
H	-2.376412000	1.777706000	0.504864000
H	-1.858769000	0.502790000	1.612897000
H	-3.213541000	0.213457000	0.525335000

CB7•**7** E(RB3LYP) = -4679.73571606

N	4.785937000	2.709110000	-1.139105000
C	4.789461000	3.329238000	0.160579000
C	3.713168000	4.452922000	0.031104000
N	3.065036000	4.138264000	-1.227792000
C	3.795324000	3.214155000	-1.972987000
N	2.920569000	4.299497000	1.222682000
C	3.277678000	3.179270000	1.964084000
N	4.295717000	2.533038000	1.265718000

C	1.941638000	5.248552000	1.689870000
N	0.646518000	5.154463000	1.050136000
C	-0.506101000	5.017682000	1.822581000
N	-1.590630000	5.277512000	0.990500000
C	-1.194192000	5.649662000	-0.342822000
C	0.360420000	5.759209000	-0.238461000
N	-1.383836000	4.659036000	-1.386648000
C	-0.200926000	4.392920000	-2.072442000
N	0.820902000	5.049881000	-1.401332000
C	-2.954689000	5.193712000	1.456984000
N	-3.702339000	4.036810000	1.019319000
C	-3.964228000	2.960671000	1.859914000
N	-4.770491000	2.071573000	1.150149000
C	-5.277048000	2.662943000	-0.074861000
C	-4.360886000	3.913424000	-0.255233000
N	-5.070440000	1.919183000	-1.289587000
C	-4.028708000	2.427539000	-2.056170000
N	-3.530881000	3.538447000	-1.383266000
C	-5.453520000	0.999235000	1.838389000
N	-5.173843000	-0.316191000	1.321810000
C	-4.368869000	-1.213038000	2.011525000
N	-4.400603000	-2.416250000	1.313264000
C	-5.378913000	-2.410133000	0.246671000
C	-5.847694000	-0.917151000	0.197927000
N	-4.893110000	-2.652587000	-1.090612000
C	-4.992728000	-1.535839000	-1.911990000
N	-5.436154000	-0.482185000	-1.119062000
C	-3.903251000	-3.625337000	1.921414000
N	-2.769580000	-4.202111000	1.241396000
C	-1.587409000	-4.446929000	1.933205000
N	-0.809013000	-5.270743000	1.128597000
C	-1.448319000	-5.593577000	-0.119073000
C	-2.897414000	-5.047589000	0.068883000
N	-0.989003000	-4.880047000	-1.298627000
C	-2.051431000	-4.313848000	-2.001193000
N	-3.172368000	-4.386903000	-1.179075000
C	0.468117000	-5.777509000	1.563240000
N	1.613355000	-5.087539000	1.023627000
C	2.480654000	-4.367608000	1.835389000
N	3.549696000	-3.971733000	1.040196000
C	3.515659000	-4.571905000	-0.275659000
C	2.135781000	-5.307423000	-0.303338000
N	3.438865000	-3.670059000	-1.395702000
C	2.238574000	-3.774106000	-2.092246000

N	1.432591000	-4.668223000	-1.392439000
C	4.725748000	-3.363775000	1.615484000
N	4.994320000	-2.016690000	1.175810000
C	5.670150000	-1.659993000	-0.042621000
C	5.909106000	-0.125674000	0.117804000
N	5.029292000	0.222500000	1.222131000
C	4.610609000	-0.908122000	1.919954000
N	5.572948000	0.397509000	-1.179104000
C	4.988150000	-0.555729000	-2.006453000
N	4.901654000	-1.735324000	-1.273612000
O	4.665815000	-0.398445000	-3.166297000
C	4.578746000	-2.976850000	-1.940132000
C	5.770576000	1.761162000	-1.605624000
C	5.080963000	1.500310000	1.895977000
O	4.058916000	-0.926407000	3.001674000
O	2.352911000	-4.165202000	3.026564000
O	1.970976000	-3.232059000	-3.144751000
C	0.237414000	-5.212871000	-1.989754000
O	-1.306552000	-4.059756000	3.049345000
O	-2.020837000	-3.881962000	-3.135366000
C	-4.478807000	-3.938402000	-1.602615000
O	-3.791904000	-1.002436000	3.059238000
O	-4.780133000	-1.499728000	-3.107034000
C	-5.840547000	0.773383000	-1.707148000
O	-3.602107000	2.842930000	3.012564000
O	-3.658389000	2.008156000	-3.134331000
C	-2.650720000	4.462623000	-2.051217000
O	-0.554702000	4.769816000	3.009850000
O	-0.090549000	3.753196000	-3.099065000
C	2.172937000	5.058756000	-1.894559000
O	2.821388000	2.853398000	3.040678000
O	3.630858000	2.939993000	-3.143988000
H	2.347329000	6.262625000	1.548500000
H	1.780322000	5.057979000	2.753342000
H	-3.493186000	6.095397000	1.134192000
H	-2.910799000	5.155031000	2.547683000
H	-5.116350000	1.023157000	2.877285000
H	-6.540783000	1.169996000	1.794242000
H	-3.574873000	-3.374325000	2.932867000
H	-4.719638000	-4.365467000	1.965609000
H	0.527890000	-6.842040000	1.293916000
H	0.512844000	-5.663325000	2.648637000
H	4.564914000	-3.331485000	2.695376000
H	5.606241000	-3.981049000	1.385207000

H	4.677043000	1.352782000	2.900436000
H	6.130714000	1.832767000	1.961292000
H	5.796357000	3.710492000	0.391141000
H	4.140714000	5.466878000	-0.008649000
H	0.729262000	6.796273000	-0.258860000
H	-1.681080000	6.594132000	-0.630867000
H	-4.916007000	4.836456000	-0.483027000
H	-6.343473000	2.911279000	0.037361000
H	-6.936838000	-0.797874000	0.308092000
H	-6.191232000	-3.114936000	0.482904000
H	-3.644106000	-5.836158000	0.250017000
H	-1.406591000	-6.679914000	-0.293036000
H	2.220651000	-6.388539000	-0.496276000
H	4.375184000	-5.249518000	-0.398543000
H	6.599867000	-2.241009000	-0.140491000
H	6.949030000	0.131619000	0.370593000
H	2.143603000	4.762647000	-2.945542000
H	2.566245000	6.083297000	-1.804687000
H	-3.155966000	5.438215000	-2.154055000
H	-2.441553000	4.048164000	-3.040272000
H	-6.897415000	0.965407000	-1.470539000
H	-5.713732000	0.668373000	-2.786995000
H	-5.216402000	-4.696871000	-1.305025000
H	-4.455129000	-3.847571000	-2.690893000
H	0.336984000	-6.310283000	-2.044999000
H	0.155934000	-4.798365000	-2.997208000
H	5.449540000	-3.651248000	-1.902902000
H	4.349687000	-2.725159000	-2.978293000
H	5.722338000	1.763365000	-2.696777000
H	6.763156000	2.092468000	-1.272058000
C	0.225158000	-1.549310000	-0.225066000
C	1.529170000	-0.876147000	0.156904000
C	1.167267000	0.333110000	1.103355000
C	-0.447957000	-0.715831000	-1.035872000
C	0.418932000	1.315908000	0.136983000
C	0.421089000	0.527048000	-1.220360000
C	0.181952000	0.055903000	2.209541000
C	-0.951162000	1.604481000	0.799959000
C	-0.960923000	0.731052000	2.029775000
C	1.830539000	-0.104446000	-1.149600000
H	2.095938000	0.753787000	1.502809000
H	0.985562000	2.242738000	-0.005034000
C	0.511898000	-0.887837000	3.322648000
H	-1.792957000	1.391379000	0.127775000

H	-1.049450000	2.662163000	1.066950000
H	2.046931000	-0.749753000	-2.004111000
H	2.626148000	0.641914000	-1.035157000
C	-1.828062000	-0.866975000	-1.582625000
H	-1.814581000	0.678889000	2.699214000
H	-0.314786000	-0.970369000	4.035235000
H	1.414938000	-0.561483000	3.854393000
H	0.726379000	-1.895383000	2.944063000
H	2.310867000	-1.529909000	0.545767000
H	0.192752000	1.141014000	-2.095149000
H	-2.336111000	-1.714358000	-1.114246000
H	-1.824428000	-1.026463000	-2.667328000
H	-2.430129000	0.030625000	-1.404043000
H	-0.153318000	-2.467562000	0.211761000

CB7•**8** E(RB3LYP) = -4679.73714376

N	3.710208000	3.715322000	-1.180787000
C	3.694626000	4.477571000	0.048139000
C	2.274186000	5.134485000	0.049738000
N	1.697788000	4.698439000	-1.200371000
C	2.578144000	3.934488000	-1.958227000
N	1.661062000	4.616495000	1.252389000
C	2.541337000	3.853530000	2.012717000
N	3.714106000	3.729063000	1.279699000
C	0.480948000	5.193431000	1.851642000
N	-0.759620000	4.797137000	1.233958000
C	-1.756702000	4.157959000	1.965150000
N	-2.911496000	4.184359000	1.194634000
C	-2.761144000	4.961178000	-0.011775000
C	-1.258828000	5.391660000	0.017043000
N	-2.882753000	4.250507000	-1.261885000
C	-1.710336000	4.271183000	-2.009030000
N	-0.728539000	4.863109000	-1.218721000
C	-4.186524000	3.765937000	1.722947000
N	-4.722840000	2.567321000	1.126218000
C	-4.909700000	1.420744000	1.887249000
N	-5.623916000	0.519892000	1.105648000
C	-6.021506000	1.073801000	-0.165730000
C	-5.473358000	2.541672000	-0.110992000
N	-5.389404000	0.531668000	-1.348847000
C	-4.719345000	1.496046000	-2.089260000
N	-4.725712000	2.664401000	-1.339328000
C	-6.018847000	-0.773474000	1.611138000
N	-5.238547000	-1.890624000	1.130732000
C	-4.272534000	-2.511316000	1.914719000
N	-3.746662000	-3.559036000	1.166299000
C	-4.485513000	-3.786919000	-0.058023000

C	-5.442108000	-2.551615000	-0.134214000
N	-3.735701000	-3.709976000	-1.286331000
C	-4.053305000	-2.588291000	-2.043029000
N	-4.984739000	-1.855020000	-1.316325000
C	-2.911675000	-4.560843000	1.784043000
N	-1.597440000	-4.693444000	1.200137000
C	-0.456596000	-4.478651000	1.966989000
N	0.622224000	-4.986626000	1.254984000
C	0.230580000	-5.636769000	0.030247000
C	-1.327884000	-5.499361000	0.028501000
N	0.627414000	-4.989982000	-1.201893000
C	-0.462454000	-4.646452000	-1.994659000
N	-1.608082000	-4.893423000	-1.248803000
C	1.941367000	-5.079337000	1.831720000
N	2.926024000	-4.201216000	1.249816000
C	3.542569000	-3.214294000	2.010056000
N	4.620812000	-2.744324000	1.272310000
C	4.790009000	-3.429399000	0.017208000
C	3.639679000	-4.490335000	0.026369000
N	4.533686000	-2.659134000	-1.180382000
C	3.501552000	-3.197424000	-1.943598000
N	2.941764000	-4.234767000	-1.209484000
C	5.543773000	-1.771910000	1.796522000
N	5.427995000	-0.450604000	1.225653000
C	6.090891000	-0.019055000	0.015286000
C	5.892681000	1.537643000	0.027101000
N	5.146975000	1.767192000	1.243099000
C	4.939511000	0.608322000	1.979027000
N	5.211095000	1.797021000	-1.220012000
C	5.029168000	0.653016000	-1.986452000
N	5.492729000	-0.420477000	-1.237202000
O	4.596008000	0.607278000	-3.119934000
C	5.504648000	-1.761897000	-1.766393000
C	4.899544000	3.107120000	-1.735091000
C	4.878343000	3.065107000	1.810465000
O	4.469203000	0.541009000	3.096895000
O	3.234213000	-2.872576000	3.134318000
O	3.188535000	-2.862060000	-3.068543000
C	1.949095000	-5.112504000	-1.774331000
O	-0.416112000	-3.981235000	3.075153000
O	-0.421994000	-4.247274000	-3.141637000
C	-2.927698000	-4.779056000	-1.819094000
O	-3.980702000	-2.222927000	3.057992000
O	-3.636528000	-2.327600000	-3.153457000
C	-5.698342000	-0.755148000	-1.918130000
O	-4.568463000	1.255710000	3.040862000
O	-4.260397000	1.359798000	-3.205616000
C	-4.151228000	3.884481000	-1.847460000
O	-1.647736000	3.712417000	3.090221000
O	-1.577265000	3.888899000	-3.154042000
C	0.524111000	5.297311000	-1.790011000

O	2.339011000	3.420660000	3.129401000
O	2.407257000	3.564408000	-3.102638000
H	0.563421000	6.292585000	1.816132000
H	0.448460000	4.857132000	2.890435000
H	-4.909070000	4.587439000	1.595086000
H	-4.044340000	3.557303000	2.785917000
H	-5.895847000	-0.741561000	2.696132000
H	-7.073679000	-0.946783000	1.357210000
H	-2.775691000	-4.265089000	2.826899000
H	-3.422580000	-5.536984000	1.735148000
H	2.290474000	-6.120005000	1.740623000
H	1.852729000	-4.807189000	2.886005000
H	5.336824000	-1.679298000	2.865282000
H	6.570216000	-2.136043000	1.641284000
H	4.703777000	2.918412000	2.878988000
H	5.755453000	3.710480000	1.656852000
H	4.516732000	5.209863000	0.042231000
H	2.302322000	6.234810000	0.086729000
H	-1.118160000	6.483869000	0.048078000
H	-3.461097000	5.811489000	0.002502000
H	-6.268271000	3.302788000	-0.081636000
H	-7.116145000	1.020788000	-0.269497000
H	-6.502604000	-2.824682000	-0.246815000
H	-5.017414000	-4.749512000	-0.002898000
H	-1.852547000	-6.464210000	0.109808000
H	0.578277000	-6.681744000	0.032709000
H	4.002732000	-5.529934000	0.042557000
H	5.795602000	-3.874958000	-0.035063000
H	7.148005000	-0.324295000	0.040692000
H	6.839595000	2.097715000	0.059100000
H	0.513112000	5.016271000	-2.845531000
H	0.599064000	6.393064000	-1.694878000
H	-4.865946000	4.705756000	-1.689276000
H	-3.980870000	3.737958000	-2.916618000
H	-6.780586000	-0.939404000	-1.831618000
H	-5.408661000	-0.718282000	-2.971174000
H	-3.461890000	-5.729782000	-1.671461000
H	-2.797759000	-4.585349000	-2.886342000
H	2.292444000	-6.153138000	-1.660167000
H	1.861796000	-4.863627000	-2.834461000
H	6.509738000	-2.190051000	-1.631634000
H	5.272298000	-1.683362000	-2.831092000
H	4.731743000	2.995545000	-2.808947000
H	5.755431000	3.774684000	-1.555418000
C	0.945968000	-1.329974000	0.742513000
C	1.357033000	0.085326000	1.109393000
C	0.076285000	0.994846000	0.924667000
C	0.727539000	-1.354578000	-0.581084000
C	-0.136770000	0.985638000	-0.622929000
C	1.006256000	0.037683000	-1.144520000
C	-1.210511000	0.449951000	1.496761000

C	-1.591021000	0.514626000	-0.862897000
C	-2.091386000	0.181096000	0.521257000
C	2.132356000	0.472927000	-0.169446000
H	0.302628000	1.991673000	1.324163000
H	0.032690000	1.975752000	-1.059597000
C	-1.361879000	0.213892000	2.966264000
H	-1.642105000	-0.346649000	-1.544291000
H	-2.187910000	1.300562000	-1.340948000
H	3.041833000	-0.118686000	-0.299744000
H	2.370492000	1.540034000	-0.235785000
C	1.254621000	0.122526000	-2.640239000
H	0.738656000	-2.108185000	1.466890000
H	0.324551000	-2.170699000	-1.170582000
H	-3.069413000	-0.259935000	0.689293000
H	-2.340065000	-0.202420000	3.215795000
H	-1.231147000	1.152503000	3.519281000
H	-0.589492000	-0.482391000	3.321629000
H	1.860990000	0.210722000	2.069049000
H	0.353160000	-0.170360000	-3.193174000
H	2.065701000	-0.541929000	-2.951202000
H	1.515346000	1.142846000	-2.940415000

References

[1] J. Krupka and J. Pašek, *Petroleum & Coal* **2010**, *52*, 227-234.

[2] T. Laurens, D. Nicole, P. Rubini, J. Lauer, M. Matlengiewicz and N. Henzel, *Magn. Reson. Chem.* **1991**, *29*, 1119-1129.

[3] F. Schmit-Quilès, D. Nicole and J. C. Lauer, *Analyst* **1994**, *119*, 1731-1740.
